# Progression of ocean interior acidification over the industrial era

**DOI:** 10.1126/sciadv.ado3103

**Published:** 2024-11-27

**Authors:** Jens D. Müller, Nicolas Gruber

**Affiliations:** Environmental Physics, Institute of Biogeochemistry and Pollutant Dynamics, ETH Zurich, Zurich, Switzerland.

## Abstract

Ocean acidification driven by the uptake of anthropogenic CO_2_ represents a major threat to ocean ecosystems, yet little is known about its progression beneath the surface. Here, we reconstruct the history of ocean interior acidification over the industrial era on the basis of observation-based estimates of the accumulation of anthropogenic carbon. Across the top 100 meters and from 1800 to 2014, the saturation state of aragonite (Ω_arag_) and pH = −log[H^+^] decreased by more than 0.6 and 0.1, respectively, with nearly 50% of the progression occurring over the past 20 years. While the magnitude of the Ω_arag_ change decreases uniformly with depth, the magnitude of the [H^+^] increase exhibits a distinct maximum in the upper thermocline. Since 1800, the saturation horizon (Ω_arag_ = 1) shoaled by more than 200 meters, approaching the euphotic zone in several regions, especially in the Southern Ocean, and exposing many organisms to corrosive conditions.

## Teaser

The rapid downward progression of anthropogenic carbon accelerates ocean interior acidification and threatens marine biota.

## INTRODUCTION

The ocean now absorbs around a quarter of the annual anthropogenic CO_2_ emission (*1*–*3*). While this net uptake of anthropogenic CO_2_ (C_ant_) mitigates climate change, it also acidifies the world’s most voluminous ecosystem (*4*–*6*). Ocean Acidification (OA) comprises multiple changes in the marine CO_2_ system, including the increase of the proton concentration ([H^+^]) leading to a reduction of pH (−log[H^+^]), as well as a decline of the carbonate ion concentration, which lowers the saturation state (Ω) of carbonate minerals (*7*). These changes are expected to have substantial impacts on the fitness of many organisms affecting also ecosystem functioning (*5*, *8*–*10*).

So far, most OA studies have focused exclusively on the surface ocean, although it is well established that the downward transport of C_ant_ acidifies the ocean also at depth. Driven by the downward transport of C_ant_ (*11*–*16*), ocean interior acidification signals penetrate by now several hundred to thousands of meters deep into the ocean, with rates of change that in some regions exceed those at the surface (*17*–*19*). This subsurface amplification can be caused by naturally acidified conditions in the ocean interior due the accumulation of remineralized dissolved inorganic carbon (DIC), which results in a higher sensitivity of some CO_2_ system parameters to a given change in DIC (*17*, *18*). Acidification in the ocean interior is of particular concern, because it is home to many OA-sensitive organisms, such as pteropods (*20*, *21*) or the highly diverse organisms inhabiting the sea floor, including cold water corals (*22*, *23*). It has been speculated that some of these organisms might be more sensitive to OA than those living at the surface (*24*, *25*).

Yet, our current quantitative understanding of the time history and progression of ocean interior acidification is not very well developed. This is largely a consequence of the dearth of observations that limit the extension of the surface ocean OA studies to depth. Notable exceptions are local studies based on time series stations (*26*–*28*) and repeat hydrography sections (*29*–*36*). All of these studies find acidification rates in the surface mixed layer of the ocean that primarily reflect the rate of increase of atmospheric CO_2_ over the observational period (*37*), causing a pH decline of ~0.002 year^−1^ and a reduction of the saturation state of aragonite (Ω_arag_) by ~0.001 year^−1^. Over the 40-year extension of the longest time series records, this corresponds to a pH decline approaching 0.1 (equivalent to an [H^+^] increase of more than 30%) and a drop of Ω_arag_ by >0.3. In some study regions, acidification rates below the mixed layer were found to be more variable than at the surface. This subsurface variability tends to occur along isopycnals (*29*) and to be more pronounced in shorter time series.

Although the main driver of ocean interior acidification in these studies is the accumulation of C_ant_, the elevated variability of interior acidification rates indicates that changes in the ocean’s pH or Ω can also occur because of changes in the natural carbon cycle (*3*) altering either DIC or alkalinity (TA) or both (*38*). These changes can be driven by water mass redistributions or changes in the ocean’s biological pumps, namely, changes in the production and/or remineralization of organic matter or changes in the production and/or dissolution of mineral calcium carbonate (*39*). Here, we focus exclusively on ocean interior acidification driven by C_ant_. This choice is based on three reasons: First, it can be expected that under exponentially growing atmospheric CO_2_ and over multidecadal to centennial timescales, changes in the natural carbon cycle have a lower impact on OA rates compared to the accumulation of C_ant_ (*29*). This is well supported by local attribution studies conducted so far (*26*, *27*, *29*, *36*), which suggest that between 60 to 100% of the observed decadal changes in pH and/or Ω at depth are due to the downward transport and subsequent accumulation of C_ant_. Second, this focus is consistent with the original coinage of the term “Ocean Acidification,” which was meant to reflect only the change in the ocean’s chemistry driven by the uptake of anthropogenic CO_2_ (*40*). Third, we have global observation–based estimates available of the accumulation of C_ant_ over the period 1800 until 2014 (*14*, *15*), permitting us to reconstruct the history of OA across the whole ocean interior over the entire industrial period.

The first such observation-based approach to reconstruct ocean interior acidification was published more than two decades ago (*41*), using a global scale estimate of the C_ant_ accumulation until 1994 (*14*). The authors focused on the shoaling of the aragonite saturation horizon (Ω_arag_ = 1) along meridional sections between preindustrial times and 1994. A unexpected finding was that the aragonite saturation horizon in the North Atlantic Ocean (20° to 50°N) had remained largely unchanged since preindustrial times. However, this conclusion was derived from a single section located in the eastern North Atlantic, where C_ant_ does not penetrate as deep as in the western part of the basin. Only recently was this early study extended to account for the time evolving nature of ocean interior acidification and lateral gradients within an ocean basin. This was achieved for the Pacific Ocean (*19*) by taking advantage of the C_ant_ reconstructions from multiple hydrography sections that were reoccupied more than twice over the past decades. This study documented the progression of ocean interior acidification and found strong spatial gradients including a subsurface intensification of the pH decline located in the oxygen minimum zone along the coast of South America (*19*). This subsurface intensification of ocean interior acidification was recently also assessed globally (*17*) and found to be particularly strong for the increase in [H^+^]. But so far, no global observation–based reconstruction of ocean interior acidification trends has been undertaken to put the regional section–based results in a global context and at the same time document in a consistent way the progression of ocean interior acidification over the entire industrial era.

Numerical ocean models provide spatiotemporally resolved OA fields and extend observation-based assessments backward and forward in time (*6*, *42*). This potential has been leveraged to demonstrate that surface OA has accelerated rapidly over the recent decades reflecting the exponential growth in atmospheric CO_2_ (*41*) and that subsurface pH changes are expected to exceed surface changes throughout much of the ocean by the end of the century (*6*). However, substantial shortcomings in simulating the marine CO_2_ system in the ocean interior (*43*, *44*), leading to strong biases in the local sensitivities to OA, especially at depth, call for observation-based reconstructions of ocean interior acidification.

The interior ocean dimension of OA is also not reflected in the planetary boundaries concept that was designed to determine safe operating spaces for humanity (*45*, *46*). The current planetary boundary of OA, based solely on surface ocean conditions, is not considered as transgressed because the global mean surface Ω_arag_ has not yet fallen below 80% of its preindustrial value (*45*, *46*). This boundary was adopted in the planetary boundary studies to represent a situation where high latitude surface waters become undersaturated with respect to aragonite (Ω_arag_ < 1) and coral reef ecosystems start to suffer from Ω_arag_ below 3 to 3.5 (*47*). Thus, this planetary boundary definition does not do justice to the impact of OA on organisms and ecosystems living beneath the surface.

The aim of this study is to provide a global observation–based history of ocean interior acidification driven by the accumulation of anthropogenic CO_2_ unraveling trends and patterns over the industrial era. We deem this important to (i) provide global context to local OA studies covering the recent past, (ii) establish a reference for the evaluation of ocean models, and (iii) inform the assessment of habitat changes for organisms populating the ocean interior. We establish our reconstruction of acidification in the ocean interior based on three dimensional fields of the accumulation of C_ant_ that have previously been obtained from observations of DIC and other biogeochemical and physical tracers using either the C* method (*14*, *48*) or the eMLR(C*) method (*15*, *49*). We combine these C_ant_ estimates with modern-day climatologies of the marine CO_2_ system, nutrients (*50*), and physical properties of the ocean (*51*, *52*). This synthesized dataset allows us to compute the state and evolution of the marine CO_2_ system from preindustrial times (~1800) to the reference years 1994, 2004, and 2014.

## RESULTS AND DISCUSSION

### Global mean history of ocean interior acidification

**Fig. 1.**
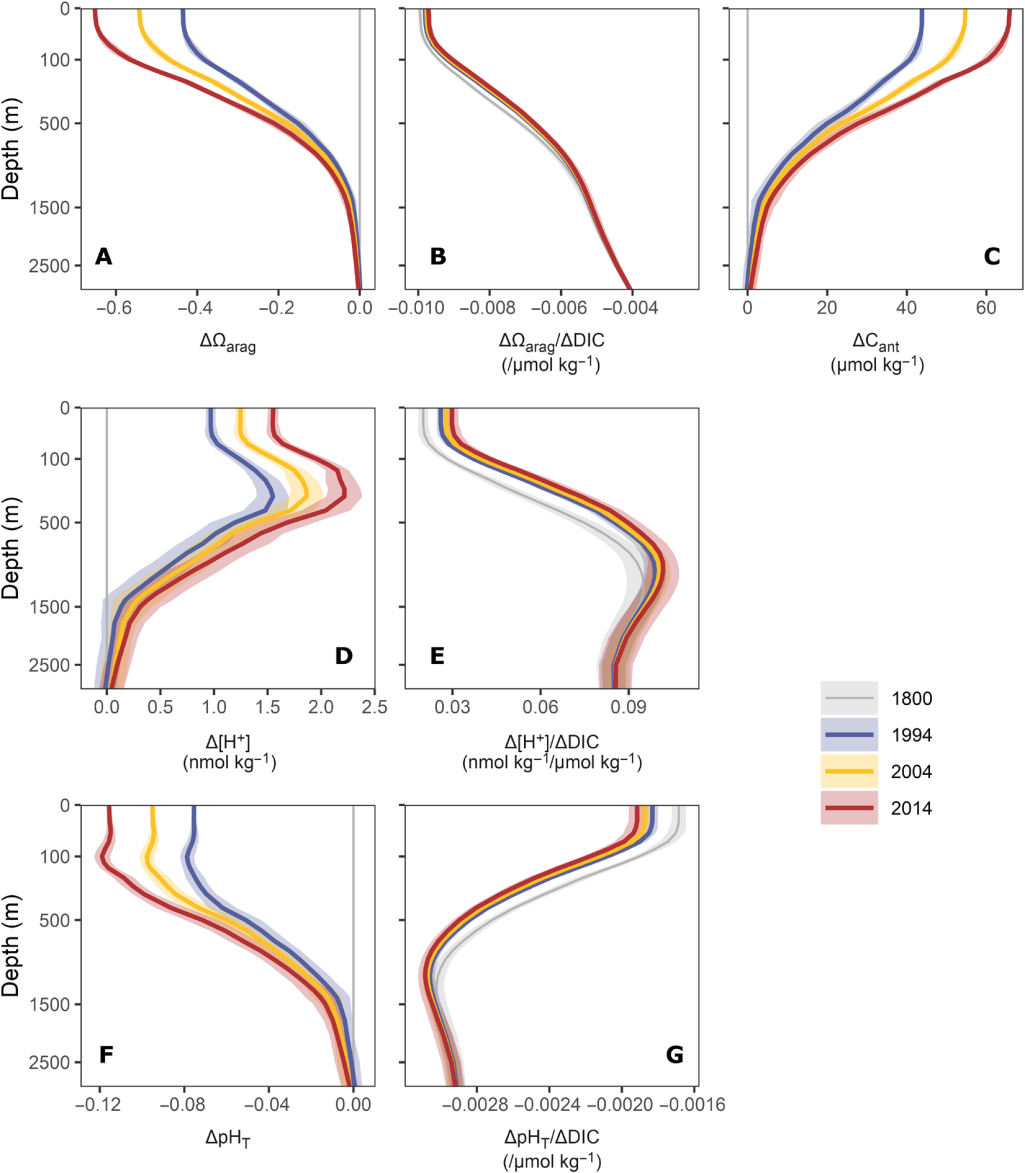
Global mean vertical profiles of the changes in the marine CO_2_ system. Progression of (**A**) changes in the saturation state of aragonite (ΔΩ_arag_), (**B**) the sensitivity of Ω_arag_ to changes in dissolved inorganic carbon (ΔΩ_arag_/ΔDIC), (**C**) changes in the anthropogenic carbon concentration (ΔC_ant_), (**D**) changes in the free proton concentration (Δ[H^+^]), (**E**) the local sensitivity of [H^+^] to changes in DIC (Δ[H^+^]/ΔDIC), (**F**) changes in pH on the total scale (ΔpH_T_), and (**G**) the local sensitivity of pH_T_ to changes in DIC (ΔpH_T_/ΔDIC). Colors distinguish changes since 1800 for the reference years 1994, 2004, and 2014 or the sensitivities for the respective years. Ribbons around lines indicate uncertainty ranges of our estimates (see Materials and Methods).

The global mean vertical profiles of the reconstructed changes in Ω_arag_, [H^+^], and pH_T_ (Fig. 1, A, D, and F) reveal the distinct history of ocean interior acidification, especially its rapid and accelerating progression deep into the ocean’s interior. Although these global mean profiles are not representative for any single place in the ocean, they illustrate the generally deep penetration of OA. In 2014, the average water column down to nearly 1500 m is measurably affected by changes. Our reconstructed changes in surface Ω_arag_ of −0.61 ± 0.01 and surface pH_T_ of −0.117 ± 0.003 over the industrial period up to 2014 (Table 1) agree well with independent estimates from a hybrid model-observation analysis (*42*), reflecting the fact that near-surface C_ant_ and thus pH and Ω_arag_ are very closely following the perturbation in atmospheric CO_2_ (*3*, *37*). Although ∆Ω_arag_ and ∆pH_T_ decrease markedly with depth, the global mean decrease in these two properties from 100 to 500 m still amounts to −0.34 ± 0.02 and −0.094 ± 0.006, respectively (Table 1), reflecting the deep-reaching and growing accumulation of anthropogenic CO_2_ in the ocean interior (Fig. 1C).

**Table 1.** Global mean state, changes, and the progression of change in the marine CO_2_ system. Results are provided for the saturation state of aragonite (Ω_arag_), the free proton concentration ([H^+^] in nanomoles per kilogram), pH on the total scale (pH_T_), dissolved inorganic carbon (DIC in micromoles per kilogram), or the anthropogenic carbon concentration (C_ant_ in micromoles per kilogram), as well as the mean depth of four isosurfaces of Ω_arga_ (in meters). Globally averaged marine surface CO_2_ (in parts per million) data from the Global Monitoring Laboratory (GML) of the National Oceanic and Atmospheric Administration (NOAA) are provided as a reference (*82*). Asterisks (*) indicate that progression of change is not reported for the depth layer of 1500 to 3000 m, because the change in the C_ant_ is below the detection limit.

		Mean state		Change		Progression
Variable	Condition	1800	2014	1800–2014	1994–2014	1994–2014/1800–1994
CO_2_	Atmosphere	282.5	395.7	113.2	39.0	52%
[H^+^]	0–100 m	5.55 ± 0.27	7.26 ± 0.33	1.72 ± 0.07	0.64 ± 0.04	59 ± 2%
Ω_arag_		3.31 ± 0.10	2.7 ± 0.10	−0.61 ± 0.01	−0.20 ± 0.01	48 ± 1%
pH_T_		8.17 ± 0.02	8.05 ± 0.03	−0.117 ± 0.003	−0.040 ± 0.001	52 ± 1%
DIC or C_ant_		2003 ± 5	2066 ± 5	63 ± 1	21 ± 1	49 ± 1%
[H^+^]	100–500 m	9.20 ± 0.37	11.24 ± 0.43	2.04 ± 0.17	0.62 ± 0.05	44 ± 3%
Ω_arag_		1.87 ± 0.05	1.53 ± 0.05	−0.34 ± 0.02	−0.10 ± 0.01	43 ± 2%
pH_T_		8.00 ± 0.02	7.90 ± 0.02	−0.094 ± 0.006	−0.029 ± 0.001	43 ± 2%
DIC or C_ant_		2135 ± 4	2175 ± 4	40 ± 2	12 ± 1	43 ± 2%
[H^+^]	500–1500 m	12.81 ± 0.57	13.58 ± 0.60	0.78 ± 0.19	0.28 ± 0.07	57 ± 14%
Ω_arag_		0.98 ± 0.05	0.90 ± 0.05	−0.08 ± 0.01	−0.02 ± 0.01	47 ± 5%
pH_T_		7.85 ± 0.03	7.82 ± 0.03	−0.031 ± 0.005	−0.011 ± 0.002	52 ± 9%
DIC or C_ant_		2247 ± 4	2259 ± 4	11 ± 2	4 ± 1	49 ± 7%
[H^+^]	1500–3000 m	12.36 ± 0.60	12.49 ± 0.60	0.14 ± 0.10	0.10 ± 0.04	*
Ω_arag_		0.76 ± 0.05	0.74 ± 0.04	−0.01 ± 0.01	−0.01 ± 0.01	*
pH_T_		7.86 ± 0.03	7.86 ± 0.03	−0.006 ± 0.003	−0.004 ± 0.001	*
DIC or C_ant_		2279 ± 4	2281 ± 4	2 ± 1	1 ± 1	*
Mean depth of isosurfaces	Ω_arag_ = 1	939 ± 133	728 ± 127	−222 ± 14	−63 ± 11	43 ± 8%
	Ω_arag_ = 2	298 ± 15	218 ± 14	−106 ± 4	−34 ± 2	44 ± 4%
	Ω_arag_ = 3	156 ± 7	101 ± 8	−77 ± 4	−29 ± 2	53 ± 6%
	Ω_arag_ = 4	73 ± 10	35 ± 15	−72 ± 7	−44 ± 6	57 ± 22%

A closer inspection reveals marked differences between the mean vertical profiles of ΔΩ_arag_ and those of ∆pH_T_ and Δ[H^+^] shown in Fig. 1. While the global mean profiles of ΔΩ_arag_ (Fig. 1A) closely mirror those of the changes in C_ant_ (Fig. 1C), the global mean profiles of ∆pH_T_ (Fig. 1F) and Δ[H^+^] (Fig. 1D) reveal subsurface extrema at around 300 m depth. In 2014, the peak of the subsurface maximum in Δ[H^+^] (2.22 ± 0.20 nmol kg^−1^) was about 50% higher than the corresponding change in the surface ocean (1.72 ± 0.07 nmol kg^−1^). For pH, the subsurface minimum is less strongly expressed, due to the logarithmic scale (*53*). These subsurface extrema in ∆pH_T_ and Δ[H^+^] were previously attributed to the increasing sensitivity with depth of [H^+^] (and pH_T_) to changes in DIC (*17*), that is, the sensitivity Δ[H^+^]/ΔDIC (and ΔpH_T_/ΔDIC) shown in Fig. 1 (E and G). In the top few hundred meters, these sensitivities increase more rapidly with depth than ∆C_ant_ decreases, leading to the maximum of the total change (Δ[H^+^] ≈ Δ[H^+^]/ΔDIC · ∆C_ant_) at around 300 m. In contrast, the sensitivity of Ω_arag_ to changes in DIC (ΔΩ_arag_/ΔDIC) decreases gradually with depth. As a consequence, the ΔΩ_arag_ profiles are surface-intensified compared to those of ΔC_ant_.

The acceleration of OA over the past 20 years is remarkable. Relative to the change that had occurred until 1994, the acidification progressed by around 50% between 1994 and 2014 (Fig. 1 and Table 1). This is primarily due to the near exponential growth of atmospheric CO_2_, pushing an exponentially growing amount of C_ant_ into the ocean, which then leads to an exponential growth of the accumulated C_ant_ at depth (*13*, *54*). Under the assumption of a steady-state circulation, this tight connection between uptake and transport leads to a steady-state accumulation of C_ant_ across all depths in proportion to the growth in atmospheric CO_2_ (*3*). In the global mean, the reconstructed C_ant_ follows this transient steady-state model quite well (Fig. 1C). This translates also into the ∆[H^+^], ∆pH_T_, and ΔΩ_arag_ profiles having nearly proportional changes with depth (Fig. 1 and Table 1) with only a few percent deviations from the global mean value. For ΔΩ_arag_, this proportional change over time is almost identical with that of C_ant_, whereas the proportional changes for [H^+^] and pH_T_ accelerated due to the increases of the respective sensitivities over time (Fig. 1 and fig. S6).

### Spatial patterns in ocean interior acidification trends

**Fig. 2.**
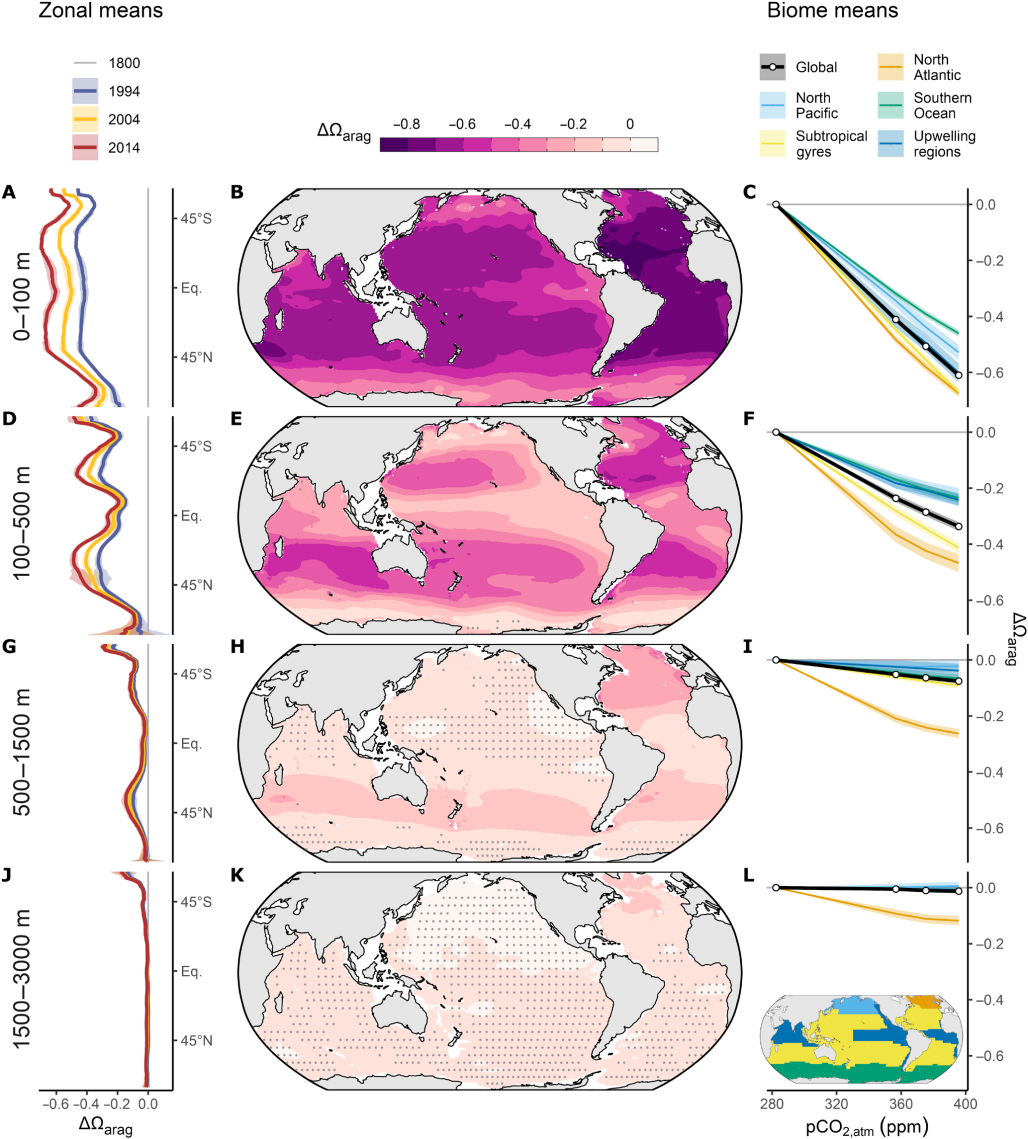
Spatial patterns in the changes of the saturation state of aragonite (ΔΩ_arag_). Acidification trends are averaged over four depth layers (panel rows), that is, 0 to 100 m, 100 to 500 m, 500 to 1500 m, and 1500 to 3000 m. (**B**, **E**, **H**, and **K**) Maps of ΔΩ_arag_ from 1800 to 2014, with stippling indicating locations where the magnitude of the acidification signal is smaller than the corresponding uncertainty. The corresponding zonal mean distributions of ΔΩ_arag_ in the left (**A**, **D**, **G**, and **J**) display changes since 1800 for the three reference years 1994, 2004, and 2014. (**C**, **F**, **I**, and **L**) Mean ΔΩ_arag_ averaged over the regions shown on the inset map (L) and plotted as a function of the increase in atmospheric CO_2_. Ribbons around lines indicate uncertainty ranges of our estimates (see Materials and Methods). Eq., equator.

**Fig. 3.**
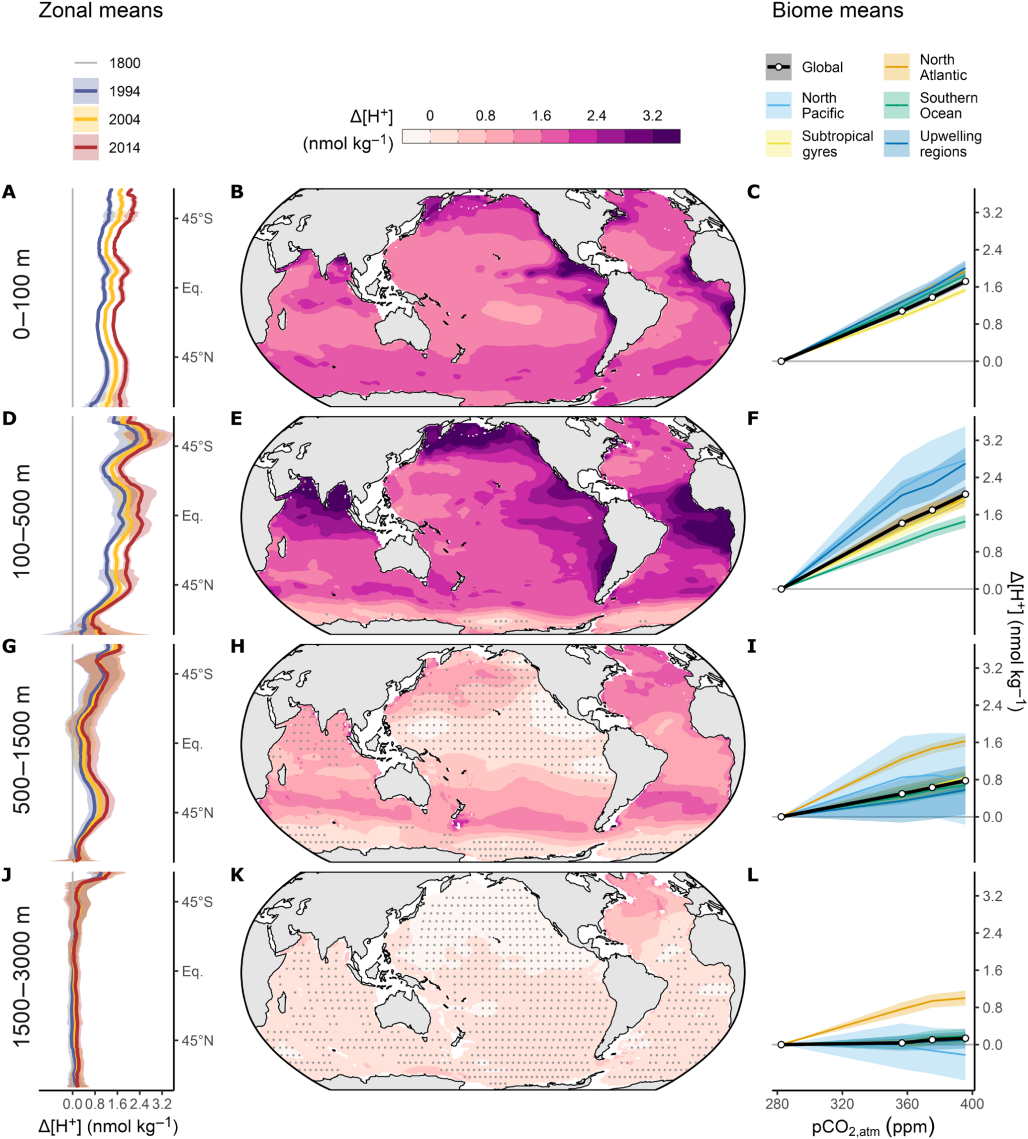
Spatial patterns in the changes of the free proton concentration (Δ[H^+^]). Figure formatting is otherwise consistent with Fig. 2, including the descriptions for (**A** to **L**).

**Fig. 4.**
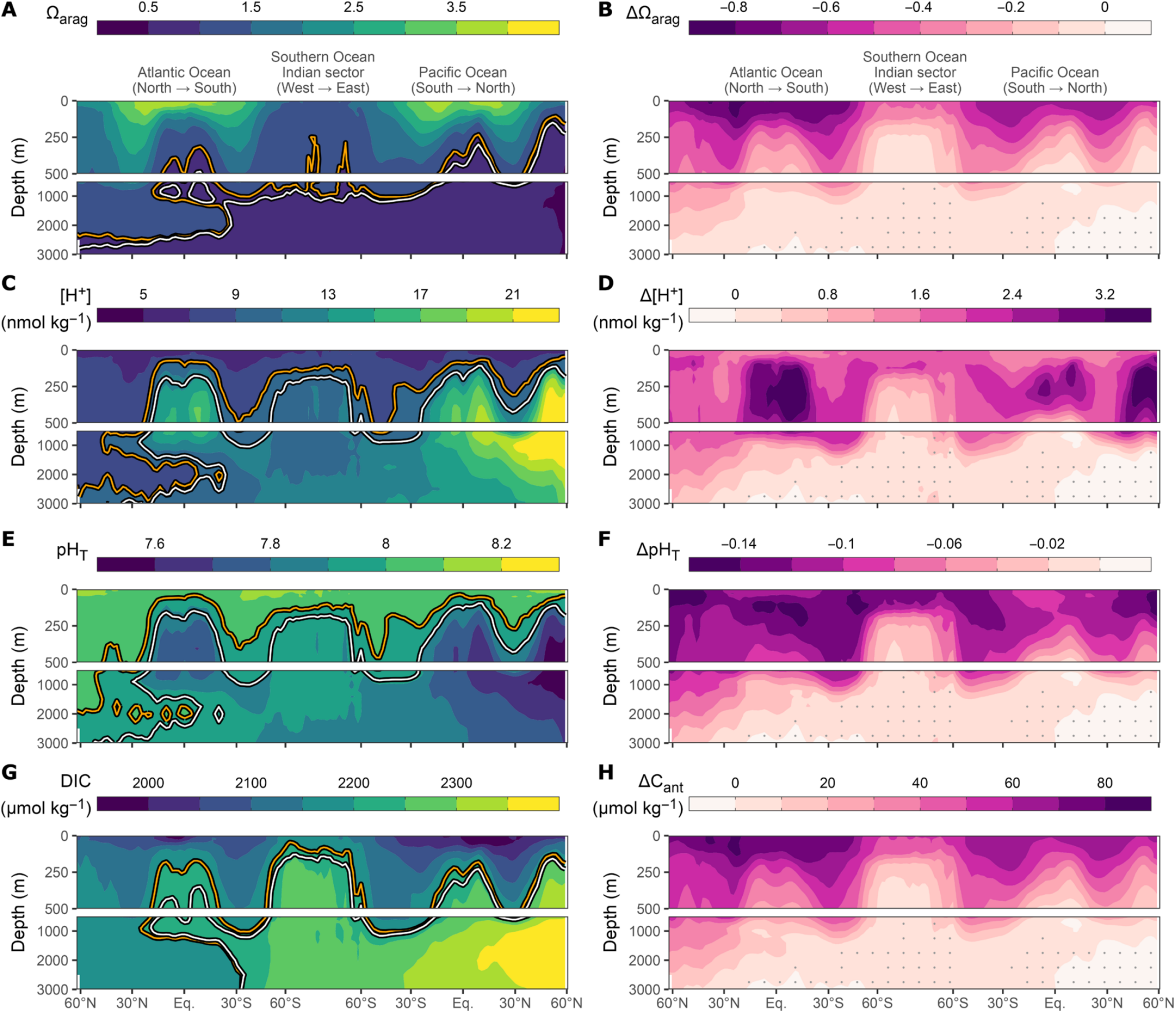
Global mean sections of the absolute state of the marine CO_2_ system in 2014 and changes from 1800 to 2014. Panel rows distinguish the saturation state of aragonite (Ω_arag_) (**A** and **B**), the free proton concentration ([H^+^]) (**C** and **D**), pH on the total scale (pH_T_) (**E** and **F**), and the anthropogenic carbon concentration (C_ant_) (**G** and **H**). Contour lines on the left indicate absolute levels of Ω_arag_ = 1, [H^+^] = 9 nmol kg^−1^, pH_T_ = 8, and DIC = 2200 μmol kg^−1^ in 2014 (orange) and 1800 (white). Stippling on the right indicates locations where the magnitude of the acidification signal is smaller than the corresponding uncertainty.

The vertical penetration of ΔΩ_arag_, Δ[H^+^], and ∆pH_T_ differs strongly between regions (fig. S2), motivating a more detailed spatial assessment (Figs. 2 to 4). As was the case for the global mean vertical profile, the spatial distribution of ΔΩ_arag_ (Figs. 2 and 4) closely resembles that of ΔC_ant_ (Fig. 4 and fig. S3). Within the top 100 m, the largest changes in Ω_arag_ occurred in the subtropical and equatorial regions, where Ω_arag_ declined by almost one unit over the industrial era until 2014 (Fig. 2B). The decline of Ω_arag_ in the top 100 m is slightly higher in the tropics and subtropics of the Atlantic compared to the Indo-Pacific. This is a result of higher accumulation rates of C_ant_ in the Atlantic, in particular from 1800 to 1994 (*14*), which itself is a consequence of the more favorable buffer factor in the Atlantic, giving it a higher uptake capacity (*37*).

In the subtropical gyres, the isolines of ΔΩ_arag_ generally penetrate deeper into the ocean compared to equatorial or high latitude regions (Fig. 4). As a consequence, the mean ΔΩ_arag_ over the depth layer 100 to 500 m reveals strong latitudinal gradients, with ΔΩ_arag_ in the center of the subtropical gyres (~30° N/S) being about twice as high as in equatorial regions or the high latitudes (Fig. 2E). The exceptionally deep penetration of ΔC_ant_ in the North Atlantic caused a decline of Ω_arag_ by more than 0.1 until 2014 even at 1500- to 3000-m depth (Figs. 2K and 4), a magnitude of change that is not found anywhere else in this depth layer.

While our reconstruction of the general patterns in ΔΩ_arag_ is consistent with previous estimates for the year 2002 (*17*), we can further unravel the temporal progression of ocean interior acidification. The latitudinal gradients in ΔΩ_arag_ vary little over time (Fig. 2, left column), as expected from the near transient steady-state development of C_ant_, which leads to a decrease in ΔΩ_arag_ that scales nearly linearly with atmospheric CO_2_ (Fig. 2, right column). An important exception is the recent decline of the acidification rate in the deep North Atlantic relative to the increase in atmospheric CO_2_ (Fig. 2L). This can be attributed to the weak C_ant_ accumulation at depth from 2004 to 2014 (*15*) shown in fig. S3.

Within the top 100 m, [H^+^] is reconstructed to have increased the most in the eastern tropical Pacific and eastern tropical Atlantic, largely a consequence of these low pH regions having the highest sensitivity Δ[H^+^]/ΔDIC (fig. S6). Apart from these regions, the highest changes in [H^+^] occurred in the high latitudes. The subsurface peak in Δ[H^+^] seen in the global mean profiles (Fig. 1D) stems largely from the equatorial upwelling regions and the North Pacific (Fig. 3). In these regions, the highest [H^+^] changes occur at around 300-m water depth and exceed 3 nmol kg^−1^ in 2014 (Fig. 4), which is almost twice as high as the global mean change in the surface layer (1.72 ± 0.07 nmol kg^−1^). Subsurface maxima in Δ[H^+^] are also widespread in the subtropical gyres. Subsurface maxima in the subtropics are not as intense as in the upwelling regions (Fig. 4) but occur several hundred meters deeper in the water column (fig. S11). A similar dislocation of the deepest and the strongest Δ[H^+^] maxima that we determine from observations has also been projected for the end of the century based on Earth System Models (*6*). Below ~1000 m, the spatial pattern in Δ[H^+^] is very similar to that of ΔΩ_arag_ in that substantial changes are only found in the North Atlantic (Fig. 4). The general subsurface intensification of Δ[H^+^] has been reported before for the year 2002 (*17*). However, the prominent role of the oxygen minimum zones in the equatorial Pacific and Atlantic was less obvious in the prior work due to the focus on individual sections at 150°W and 25°W, respectively, instead of the maps (Fig. 3E), zonal mean sections (Fig. 4D), and regionally averaged profiles (fig. S2) shown in this study. The perspectives taken here also indicate that subsurface maxima in ΔpH_T_ are emerging in upwelling regions and the North Pacific, whereas this phenomenon was postulated but not yet coherently found in 2002 (*17*).

As was the case for ΔΩ_arag_, the latitudinal gradients of Δ[H^+^] appear to change little over time (Fig. 3, left column); however, this impression is also a consequence of the major changes that were already established back in 1994. More nuances in the changes over time emerge when Δ[H^+^] is plotted versus the increase in atmospheric CO_2_ (Fig. 3, right column). Consistent with the near transient steady-state development of C_ant_, Δ[H^+^] tends to increase in proportion to the increase in atmospheric CO_2_, but deviations in the rate of ocean interior acidification that are larger than for ΔΩ_arag_ become evident in upwelling regions, the North Pacific, and the North Atlantic. These deviations have not been documented before and are related to higher changes in the sensitivity Δ[H^+^]/ΔDIC over time (fig. S6), which amplifies the temporal variability in ΔC_ant_ (fig. S3).

In summary, the spatial patterns in ocean interior acidification trends are a direct consequence of the accumulation of C_ant_ and the local sensitivities of the CO_2_ system (figs. S5, S6, and S10). These sensitivities are generally high when the absolute values of Ω_arag_ and [H^+^] are high (Figs. 4 and figs. S8 and S16). To first order, these background conditions are determined by the accumulated amount of remineralized DIC, which decreases/increases Ω_arag_/[H^+^], respectively. This prominent role of remineralized DIC in controlling ocean interior acidification rates has previously been investigated in detail (*17*, *18*, *55*), albeit without considering the progression of OA beyond 2002.

### Acidification of the seafloor and integrated across water masses

**Fig. 5.**
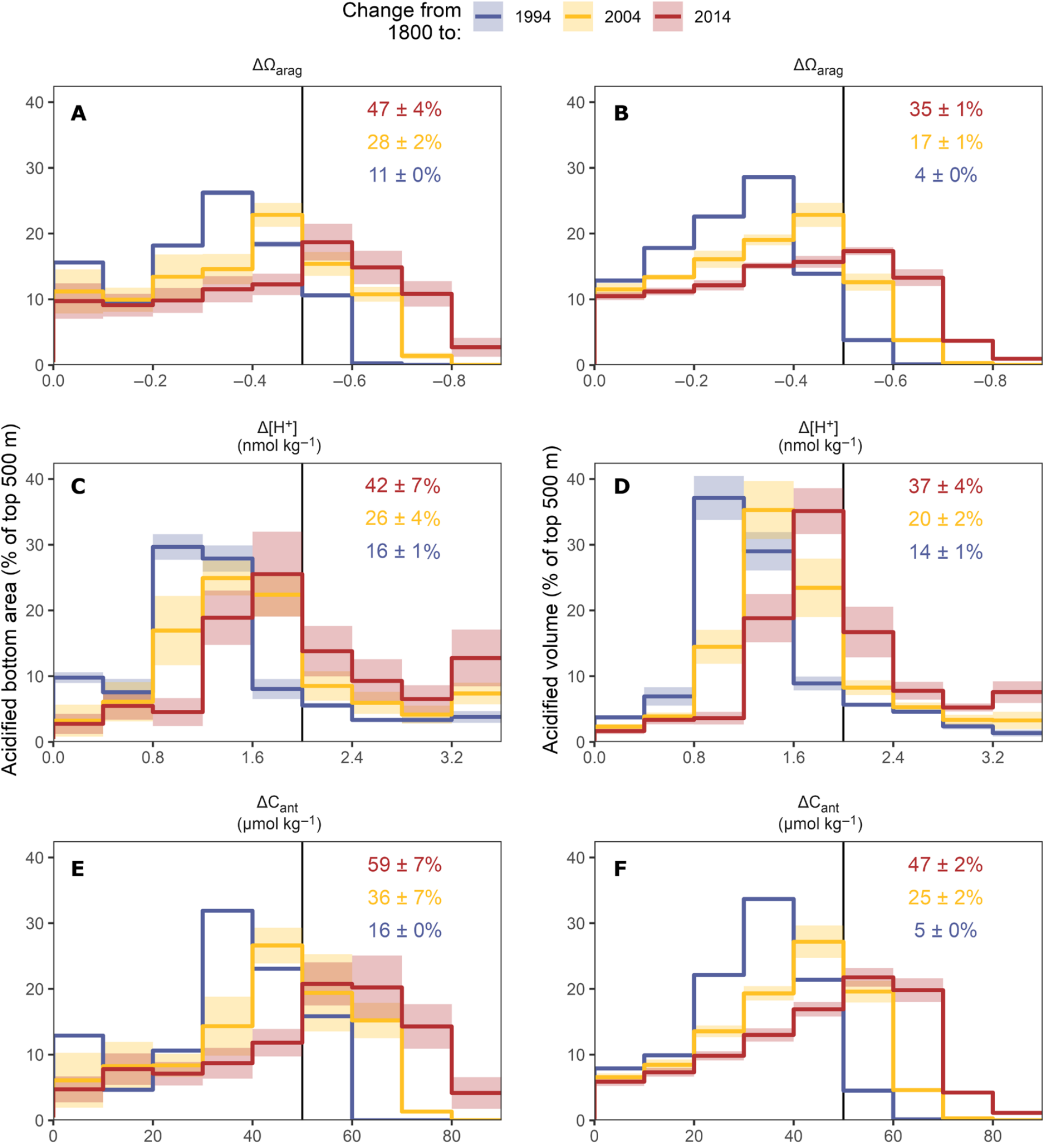
Acidified seafloor area and acidified volume fractions of the ocean. Estimates are determined over the top 500 m of the ocean for intervals of changes in (**A** and **B**) the saturation state of aragonite (ΔΩ_arag_), (**C** and **D**) the free proton concentration (Δ[H^+^]), and (**E** and **F**) the anthropogenic carbon concentration (ΔC_ant_). Colored lines distinguish fractions for 1994, 2004, and 2014, with ribbons indicating ranges of uncertainty (see Materials and Methods). Numbers in panels indicate the fraction of the total seafloor area or volume where the acidification exceeds the threshold indicated by the vertical black lines.

A region of special concern for OA is the seafloor, inhabited by a large number of benthic organisms that are sensitive to changes in seawater carbonate chemistry. To document the global progression of OA at the seafloor in general (that is irrespective of the distribution of particularly sensitive organisms), we consider the regions shallower than 500 m (see Materials and Methods and fig. S14), as acidification levels below this depth remain low, except for the North Atlantic (Fig. 4). As displayed in Fig. 5A, we find that the proportion of the seafloor that experienced a decline in Ω_arag_ by more than 0.5 (vertical black line) quadrupled over the two most recent decades of our reconstruction from 11 ± 0% in 1994 to 47 ± 4% in 2014. The strongest reduction in the affected seafloor fraction occurred in the ΔΩ_arag_ interval −0.3 to −0.4, from ~30% in 1994 to roughly 10% in 2014. The most common ΔΩ_arag_ interval in 2014 was −0.5 to −0.6, while it was −0.3 to −0.4 in 1994. These Ω_arag_ changes along the seafloor are largely mirrored by the Ω_arag_ changes in the volume fractions over the top 500 m (Fig. 5B). For example, the ocean volume affected by a decline in Ω_arag_ by more than 0.5 increased from <5% to 35 ± 1%. Furthermore, the distribution of the changes in Ω_arag_ closely mirrors the distribution of changes in C_ant_ (Fig. 5, E and F), reflecting the absence of a subsurface intensification.

Like for Ω_arag_, we find a strong increase of the seafloor (and volume) fractions that are affected by high changes in [H^+^] (Fig. 5, B and C). The fractions that experienced Δ[H^+^] ≥ 2.0 nmol kg^−1^ (roughly equivalent to ΔpH_T_ < −0.1) grew from 16 ± 1% (and 14 ± 1%) in 1994 to 42 ± 7% (and 37 ± 4%) in 2014. The strongest corresponding reduction in affected seafloor and volume fractions was identified in the Δ[H^+^] interval 0.8 to 1.2 nmol kg^−1^. The subsurface intensification of Δ[H^+^] (Fig. 4) is also reflected in different distributions of Δ[H^+^] and ΔC_ant_ at the seafloor and the affected volume distribution (Fig. 5). Below, we will discuss how acidification at the seafloor affects the conditions at known locations of cold water corals.

### The shoaling of the saturation horizon and the loss of saturated waters

**Fig. 6.**
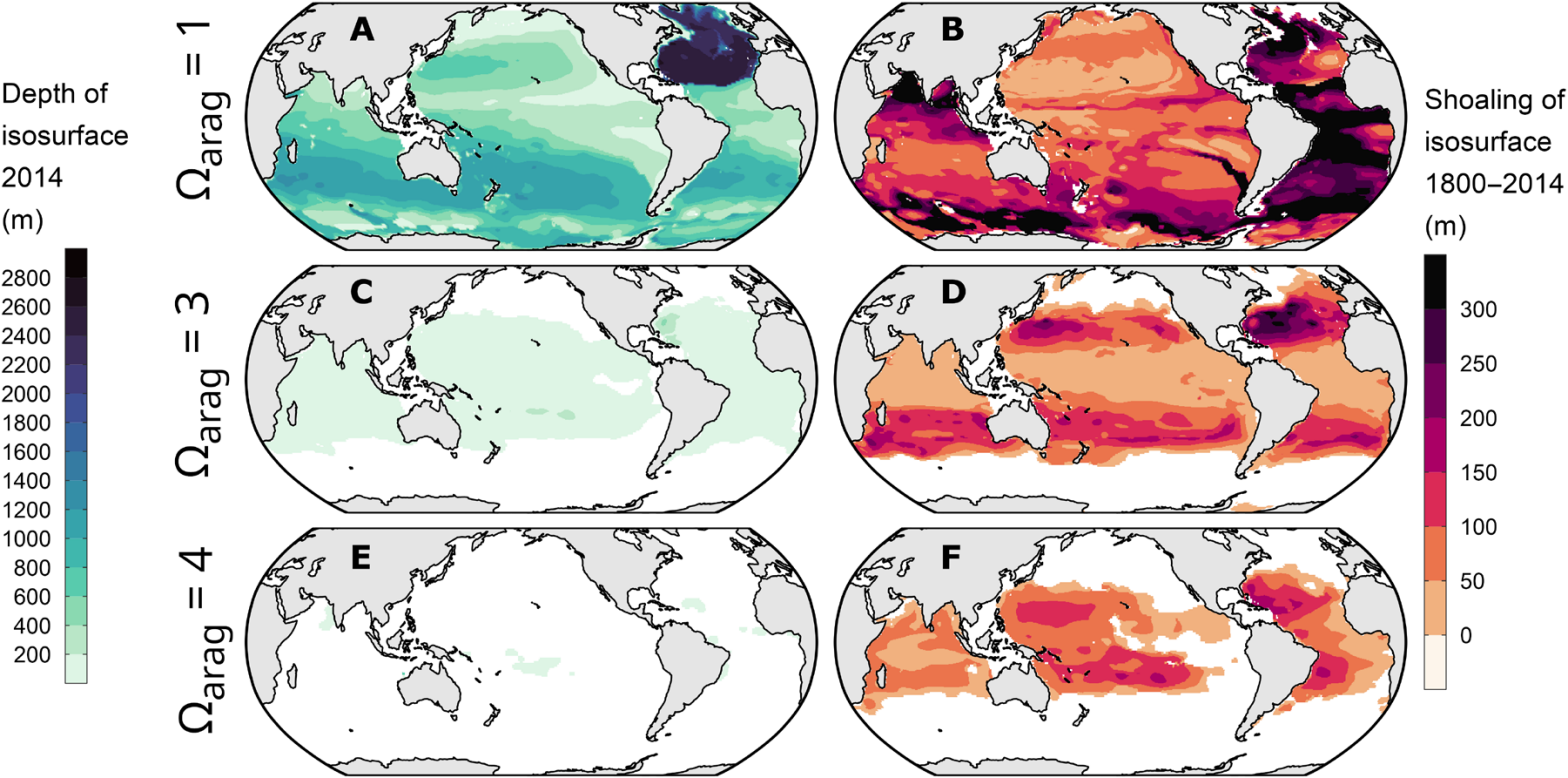
Depth of isosurfaces of the saturation state of aragonite (Ω_arag_) in 2014 and their shoaling from 1800 to 2014. Displayed are the isosurfaces for the saturation states 1 (**A** and **B**), 3 (**C** and **D**), and 4 (**E** and **F**).

**Fig. 7.**
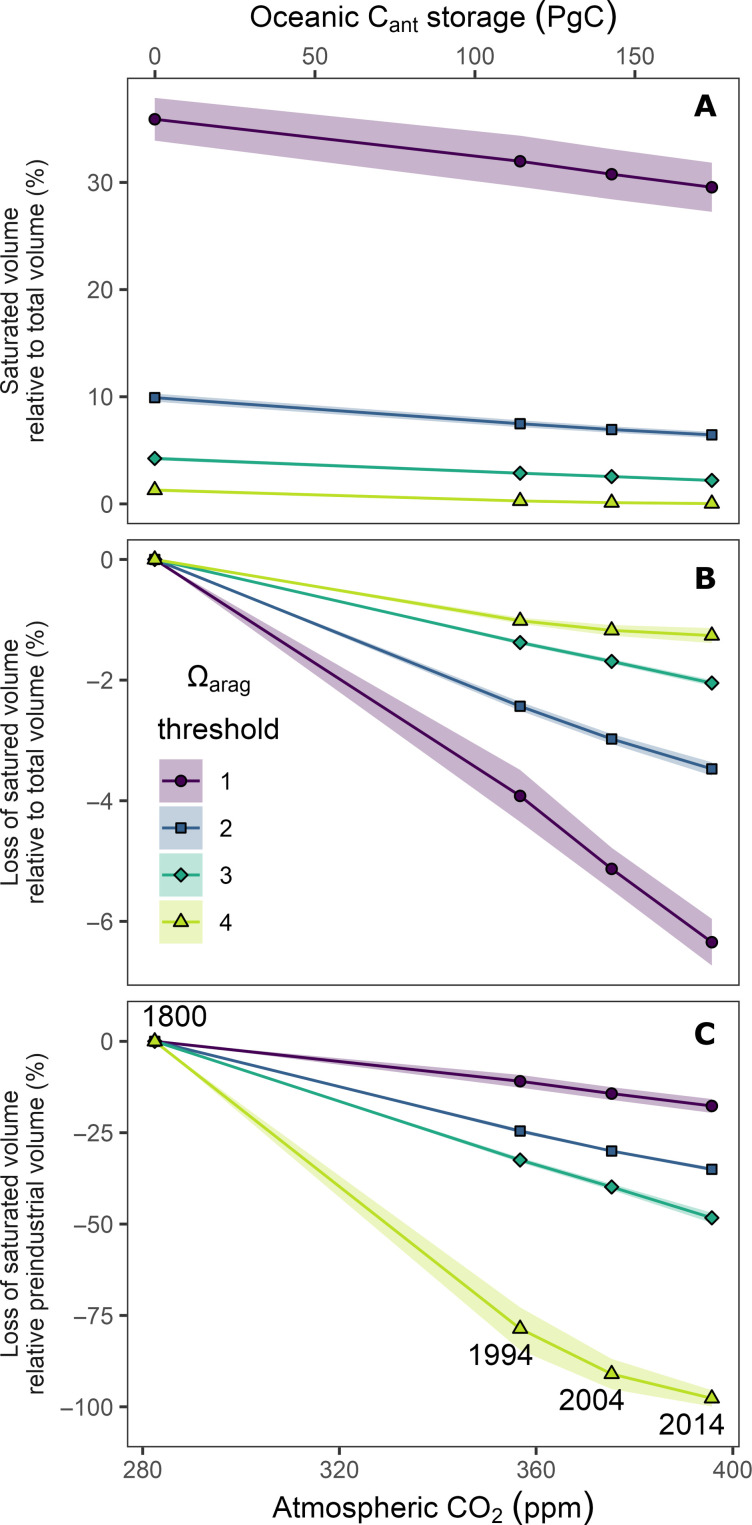
Changes in ocean volumes supersaturated with respect to aragonite for four saturation thresholds (1, 2, 3, and 4). Volume changes are displayed as a function of atmospheric CO_2_ (bottom *x* axis) and the globally integrated oceanic storage of anthropogenic carbon (C_ant_, top *x* axis). Supersaturated volumes are integrated over the top 3000 m and displayed as (**A**) the relative fraction of the volume of the upper 3000 m of the ocean, (**B**) the corresponding volume loss, and (**C**) the volume loss relative to the saturated volume in preindustrial times. Ribbons around lines indicate uncertainty ranges of our estimates (see Materials and Methods).

Further insight into the progression of ocean interior acidification can be obtained by looking at the shoaling of the saturation horizon and that of the depths of various other isosurfaces of Ω_arag_ (Figs. 4 and 6) as well as the corresponding volume loss of waters above these Ω_arag_ thresholds (Fig. 7). In consistency with previous studies, we restrict this analysis to the top 3000 m of the ocean to avoid reconstruction uncertainties of ΔC_ant_ in the deep ocean (*15*, *56*). Hence, relative volume changes are referenced to the total ocean volume above 3000 m.

An illustrative example is the shoaling of the isosurface Ω_arag_ = 4, which is considered a threshold for optimal growth of warm water corals (*57*). Back in 1800, this isosurface still existed throughout most parts of the tropical ocean between 30°N and 30°S (Fig. 6), but by 2014, it had surfaced in most regions of the world ocean except for very small remaining patches in the tropics. This near complete disappearance of waters with a mean Ω_arag_ ≥ 4 from the global ocean is consistent with independent assessments of changes of Ω_arag_ at the ocean surface (*42*). However, temporarily and locally water masses may still experience Ω_arag_ ≥ 4, for example, due to the seasonal elevation of the saturation state through primary production. Furthermore, it should be recalled that our reconstructions do not consider ocean warming, which tends to elevate the saturation state but had a much weaker impact than the C_ant_ accumulation over the past two decades (fig. S2).

The fate of water masses with Ω_arag_ above 3, which is considered as marginal to inadequate for coral growth (*57*), has followed a similar trajectory as those for the Ω_arag_ = 4 threshold. The mean depth of the isosurface Ω_arag_ = 3 shoaled over the industrial period by 77 ± 4 m to 101 ± 8 m in 2014 (Table 1 and Fig. 6), leading to a 40% reduction of the preindustrial volume of waters with Ω_arag_ > 3 (Fig. 7). The surface area of the ocean maintaining Ω_arag_ > 3 reduced only by about 20% over the industrial and is hence not strongly correlated to the shoaling of the corresponding isosurface and volume loss, suggesting that the remaining surface area of saturated conditions is a weak (or at least incomplete) indicator of the progression of OA in the ocean’s interior.

The isosurface of Ω_arag_ = 1, commonly referred to as the saturation horizon, which separates waters supersaturated in aragonite above it from undersaturated waters below it, shoaled from 940 ± 133 m in 1800 to 728 ± 127 m in 2014 (Table 1). This corresponds to a global average shoaling by 222 ± 14 m over the industrial era and exceeds previous observation-based shoaling estimates for the reference year 1994 (*41*). The uncertainty in our absolute estimates of the saturation horizons is comparably large, reflecting the mapping uncertainties in the underlying climatologies of the marine CO_2_ system (*50*). Nevertheless, the shoaling can be determined more precisely, because the bias structures in the climatologies are time-invariant and hence allow for a rather accurate determination of changes over time. The shoaling up to 2014 caused a loss of saturated water masses of 6.3 ± 0.5% of the top 3000 m ocean volume (Fig. 7), corresponding to a loss of about 20% of the preindustrial volume with Ω_arag_ ≥ 1. This is expected to have strong impacts on global biogeochemical cycles by increasing the volume of water masses that are corrosive for aragonite and hence favor the dissolution of particulate inorganic carbon (*41*).

The shoaling of these isosurfaces and the history of the loss of waters above a certain Ω_arag_ tend to scale rather linearly with the increase in atmospheric CO_2_ and the accumulation of anthropogenic CO_2_ in the ocean (Fig. 7). For example, the shoaling of the Ω_arag_ = 3 isosurface of about 80 m occurred in response to atmospheric CO_2_ going up by 107 parts per million (ppm), yielding a proportionality of ~0.7 m ppm^−1^ (Fig. 7). Hence, the current growth rate of atmospheric CO_2_ of around 2 ppm year^−1^ causes an additional shoaling of this isosurface by 1.4 m year^−1^. The global mean saturation horizon of aragonite (Ω_arag_ = 1) shoals now by 4.2 m year^−1^, corresponding to the loss of an additional 1.5% of supersaturated water masses per decade.

While the saturation horizon shoals globally relatively slowly and is still rather deep, the situation is very different in the Southern Ocean. Here, the shoaling of the saturation horizon over the industrial era is among the highest across the globe, amounting to nearly 300 m, on average (Fig. 6). The reason for this pronounced shoaling are the generally weak vertical gradients in the saturation state for Ω_arag_ ≈ 1 in the Southern Ocean (fig. S12), such that a small change in Ω_arag_ can induce a strong uplift of the saturation horizon (*58*). An even larger shoaling occurred in the regions of the Southern Ocean that represent the shallowest 10% of the saturation horizon. There, the saturation horizon migrated upward over the industrial period by more than 700 m, reaching by 2014 a mean depth of 250 ± 336 m. Hence, the surface layer of the Southern Ocean is primed to locally experience already now corrosive conditions with respect to aragonite, putting its sensitive organisms at risk (*58*, *59*).

### Impact on two groups of ecologically relevant marine organisms

We contextualize our reconstructed history of acidification in the ocean interior further by illustrating its potential impact on cold water corals and pteropods, two ecologically important groups of organisms that inhabit the ocean interior and are widely dispersed around the globe. Given the uncertainties associated with the ecological/physiological sensitivities of these two groups to OA, our results should be considered as indicative of the potential impacts at best.

**Fig. 8.**
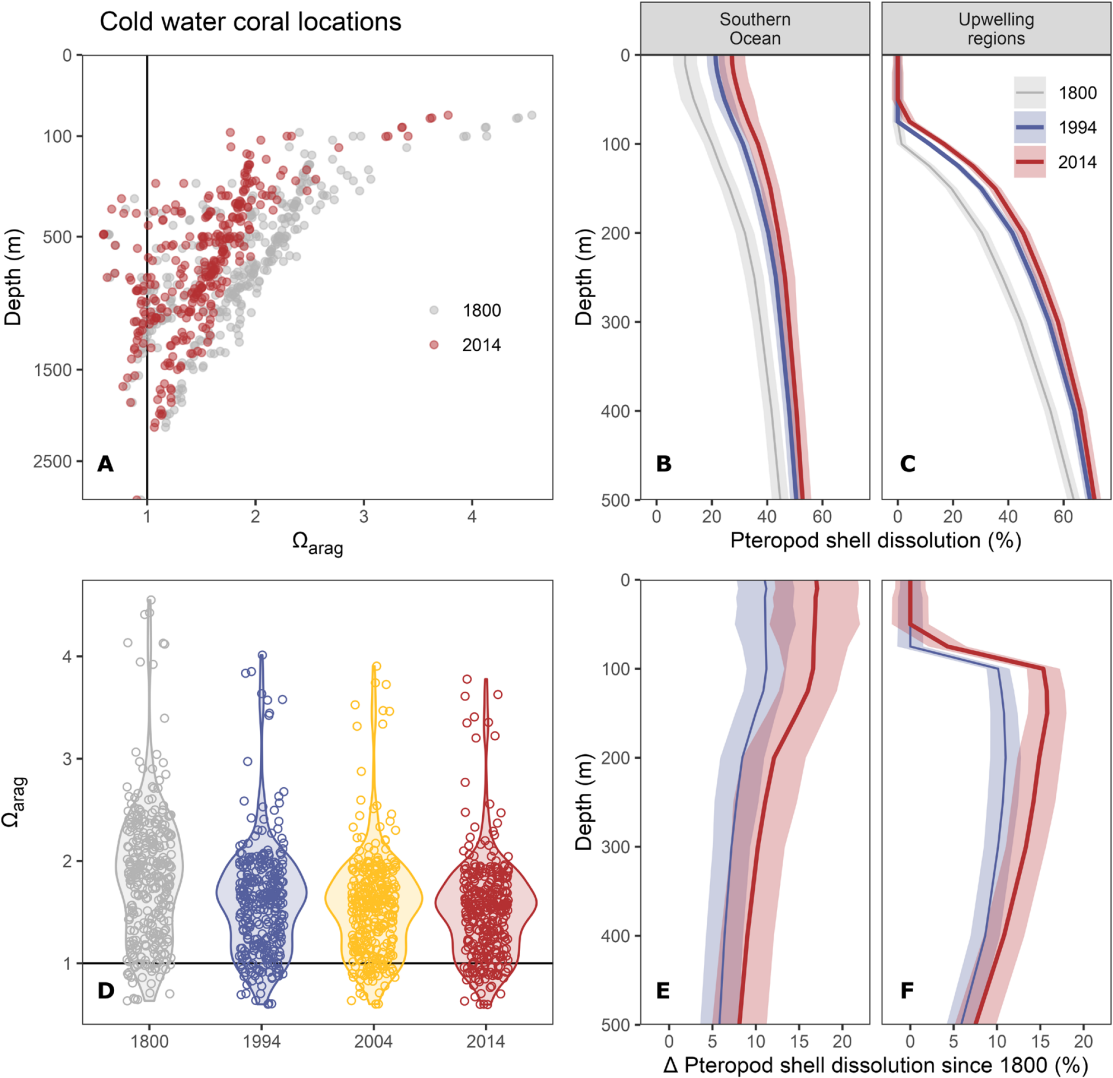
Empirically estimated impact of ocean interior acidification on benthic (cold water corals) and pelagic (pteropods) organisms. (**A** and **D**) The saturation state of aragonite (Ω_arag_) at known locations of cold water corals, following a previous model-based assessment (*23*) but building on updated occurrence data (*60*). (**B** and **C**) The vertical distribution of shell dissolution in pteropods for the Southern Ocean and upwelling regions, as well as (**E** and **F**) its increase since 1800. Shell dissolution is estimated from our Ω_arag_ reconstructions (fig. S12) based on an empirical relationship (*21*) and expressed in percent of pteropod individuals with severe shell dissolution. Ribbons around lines indicate uncertainty ranges of our estimates (see Materials and Methods).

First, we investigate the progression of ocean interior acidification at known locations of cold water corals. Their reefs form biodiversity hotspots along the edges of continental shelves and seamounts (*22*) and occur down to depths of several hundreds of meters (Fig. 8A). Here, we rely on an updated database for the global distribution of cold water corals (*60*), from which we obtained 326 unique locations of six species [*Lophelia pertusa*, *Madrepora oculata*, *Goniocorella dumosa*, *Oculina varicosa*, *Enallopsammia profunda*, and *Solenosmilia variabilis*; following (*23*)] within our study domain, including habitats found beneath 500 m. We find that 8% of these locations occurred in undersaturated conditions around 1800 (Fig. 8D), broadly consistent with previous model-based results (*23*). By 2014, this exposure almost doubled to 14%, but again without considering a minor stabilization of Ω_arag_ through warming (fig. S2). In addition, the fraction of cold water coral living in conditions characterized by Ω_arag_ < 2 increased from 63 to 92% over the industrial period. As a consequence, the range of Ω_arag_ conditions shifted downward and compressed by about 30% over the industrial period (Fig. 8D), expressed in a decline of the interquartile range from ~0.9 in 1800 to ~0.6 in 2014. This compression of Ω_arag_ conditions has been identified before in a modeling study (*61*) and bears the potential of a rather sudden transgression of large fraction of cold water coral locations into undersaturated conditions. Subsurface OA will continue to progress even under the hypothetical scenario that CO_2_ emissions immediately decline to zero, because of the redistribution of anthropogenic carbon that is already stored in the upper ocean. For example, it is predicted that the volume loss of supersaturated waters will progress for another two centuries after carbon emissions stop (*62*). This acidification in the pipeline may eventually expose cold water corals to corrosive conditions, even at locations that now provide Ω_arag_ > 1.

Second, we explore the potential impact of subsurface OA on shell dissolution in pteropods. These zooplanktonic pelagic calcifiers form an important part of the food web and drive a substantial fraction of the global carbonate export through sinking of their aragonite shells (*63*). Pteropods are susceptible to OA in the ocean interior, because the organisms migrate vertically up to several hundred meters (*64*). Our assessment relies on an empirical relationship between the fraction of pteropods affected by severe shell dissolution and environmental Ω_arag_ (*20*, *21*). While this relationship has been determined from samples in the California Current System, similar responses were identified from manipulative experiments and in situ observations across a variety of environmental conditions, including polar regions (*65*–*70*). Applying the local relationship [shell dissolution = −66.29 × ln(Ω_ar_) + 61.2] to our reconstructions of Ω_arag_ (fig. S12), we find distinct regional differences in the estimated shell dissolution and its change over time. In the Southern Ocean, the entire top 500 m of the water column were already favorable for pteropod shell dissolution in preindustrial times, with a vertical gradient from around 10% potential shell dissolution near the surface to 40% at 500 m. The potential for shell dissolution increased by around 10 to 20% over the industrial era with only a weak gradient across depth, although the reduction in Ω_arag_ is strongly surface-intensified. This difference in the vertical gradient is a consequence of the higher sensitivity of the shell dissolution on Ω_arag_ at depth, where Ω_arag_ is naturally lower. Averaged over the upwelling regions defined in our study (Fig. 2L), we find that surface Ω_arag_ remained sufficiently high to prevent shell dissolution throughout the industrial era (Fig. 8C). However, low background Ω_arag_ conditions below 100 m are strongly favorable for shell dissolution. Similar to the subsurface maxima in ΔH^+^ (fig. S2), the potential for shell dissolution in these naturally acidified conditions increases strongly over time (Fig. 8F). Our results indicate that the conditions favorable for shell dissolution of pteropods spread out over the industrial era and that the magnitude of this deterioration is highly region specific.

### Caveats and limitations

The most important caveat regarding our estimates of ocean interior acidification trends is their reconstruction solely based on the accumulation of anthropogenic carbon. The reconstructed ΔC_ant_ do not resolve local or regional changes caused by heaving or redistribution of water masses. And the reconstructed changes in Ω_arag_ and [H^+^] do not include the potential contribution of changes in DIC and TA caused by changes in the natural carbon cycle.

Despite this caveat, our surface OA trends (Figs. 1 to 3) agree remarkably well with previous estimates based on an ensemble of 14 Earth System Models (ESM) that were offset-adjusted to match observation-based patterns in sea surface temperature (SST), sea surface salinity (SSS), DIC, and TA (*42*). The model-based global mean increase of [H^+^] by ∼30% (from 6.5 to 8.5 nmol kg^−1^) from 1750 to 2010 is slightly higher than our estimate (+1.72 ± 0.13 nmol kg^−1^) due to their coverage of high OA trends in the Arctic. The good agreement of these two independent reconstructions confirms that the accumulation of C_ant_ is the prime driver for surface OA trends over the industrial era. Comparing our estimates to an entirely observation-based reconstruction of the global surface OA over the past four decades (*37*), we find good agreement in the global mean trends when those are expressed relative to the increase in atmospheric CO_2_. However, an interesting difference is that at the ocean surface slightly higher rates of change were observed in the equatorial regions of the Pacific compared to the Atlantic, whereas we find the opposite (Figs. 2 and 3). This difference can most likely be attributed to positive surface ΔC_nat_ (*37*) in the Pacific from 1982 to 2021, due to La Niña conditions (low SST and high DIC) toward the end of the period of the surface study.

Our ocean interior acidification trends are also overall consistent with direct observation-based estimates. For example, repeated pH measurements conducted in 2006 and 1991 along a meridional section in the North Pacific (P16N) revealed a shoaling of the isocline that marks C_ant_-driven pH changes of −0.01 from around 600 m at 30°N to 200 m at 50°N (*29*). This latitudinal distribution is well represented in our reconstruction (fig. S4). However, it was also found that around this isocline, pH changes driven by C_nat_ variability dominated the observed acidification. Similar variability of pH changes around the secular trend driven by the accumulation of anthropogenic carbon was also observed from repeat hydrography sections in the Atlantic (*36*) and at time series stations (*26*, *27*). It is exactly this type of variability on local to regional and interannual to decadal timescales that our global C_ant_-based reconstruction cannot resolve. The lack of such natural CO_2_-driven regional and short-term variability in our acidification estimates should also be considered when interpreting our two ecological impact assessments. Our reconstruction of the aragonite saturation state changes at cold water coral locations, for example, aims to unravel trends across two centuries, but it neglects short-term variability around these trends, which has been observed at cold water coral (CWC) locations (*71*, *72*) and might either reinforce the acidification pressure or could also create temporal acidification refugia (*73*).

### Summary and outlook

In this study, we provide a global reconstruction of the progression of C_ant_-driven acidification in the ocean interior over the industrial era. We confirm previous studies (*17*, *55*) in that the ocean interior acidification patterns differ substantially among acidification parameters, suggesting that OA impact studies should distinguish between these parameters. This conclusion holds for absolute changes in the acidification parameters (as presented in this study), whereas the spatial patterns of OA in the ocean interior are more similar across parameters when changes are expressed relative to the preindustrial state (*53*). Additional insight emerges from the time-resolved nature of our study. Over the past two decades of our reconstruction (1994–2014), the relative progress of OA was very even across depth, with an amplification of the acidification signal by about 50% compared to the level already reached in 1994. This leads to a state of ocean interior acidification in 2014 that we consider critical with respect to regional crossings of important thresholds in the ocean interior, such as a decline of the saturation state of aragonite below 3 or 1. We exemplified the potential impact of ocean interior acidification over the industrial era on two groups of environmentally relevant organisms: While we inferred a substantial elevation of pteropod shell dissolution, the exposure of cold water coral locations to undersaturated conditions doubled but remained at a low level. However, we know that ocean interior acidification has—in contrast to surface OA—a long-term commitment, that is, acidification in the ocean interior will progress for hundreds of years after emissions peak (*57*, *62*) due to the redistribution of already accumulated C_ant_ in the ocean interior. Hence, our results indicate that human CO_2_ emissions have already put ecosystems in danger that thrive hundreds or thousands of meters beneath the ocean surface.

We caveat that our reconstructions do not resolve OA driven by variability in the natural carbon cycle or other environmental drivers such as temperature. Through a comparison to previous, directly observation-based studies, we highlight that neglecting these changes is critical at local to regional and interannual to decadal timescales. Hence, we refrained from detailed assessment of the decadal variability in ocean interior acidification by comparing changes over the two recent decades, although this has been a focus of the underlying ΔC_ant_ reconstruction study. To move forward in this direction, we consider it an important next step to distinguish drivers of ocean interior acidification more scrupulously by resolving C_ant_- and natural carbon cycle–driven changes at global scale. A prerequisite for this endeavor is the availability of all required time-resolved ocean interior forcing fields. While DIC (*74*) and temperature (*75*) reconstructions are already available, we recommend prioritizing the development of a similar product for alkalinity to achieve this goal.

## MATERIALS AND METHODS

### Computation of trends and sensitivities of CO_2_ system variables based on storage changes in anthropogenic carbon

We estimate ocean interior acidification trends based on reconstructions of the anthropogenic carbon accumulation (ΔC_ant_) over the industrial era, which we combine with a present-day DIC climatology to infer the ocean interior DIC distribution at four reference years (*t*_ref_). Specifically, we determined DIC for 1994 and 2004 by adding/subtracting proportional fractions of ΔC_ant_ for the period 1994–2004 (*15*) to/from the DIC climatology centered on the year 2002 (*50*), that is, DIC(*t*_ref_) = DIC (2002) + (*t*_ref_ − 2002) × 1/10 × ΔC_ant,1994–2004_. Furthermore, we obtained DIC in 1800 by subtracting ΔC_ant_ for the period 1800–1994 (*14*) from DIC in 1994. Likewise, we added ΔC_ant_ for the period 2004–2014 to DIC in 2004 to obtain DIC in 2014.

On the basis of the four derived DIC climatologies for the reference years 1800, 1994, 2004, and 2014, we calculated the corresponding ocean interior distributions of marine CO_2_ system variables using the climatologies of salinity and temperature from the World Ocean Atlas 2018 (*51*, *52*), which were chosen for consistency with the fields used for the reconstruction of ΔC_ant_ from 1994 to 2014, as well as alkalinity (TA), silicate, and phosphate from the gridded Global Ocean Data Analysis Project (GLODAP) climatology (*50*). All CO_2_ system calculations were done with the R-package seacarb (*76*) using the CO_2_ dissociation constants updated for cold waters (*77*) in combination with the fluoride association constant (*78*) and the acidity constant of hydrogen sulfide (*79*). OA trends were obtained for all CO_2_ system variables by subtracting the value in 1800 from those in 1994, 2004, and 2014.

Our approach to reconstruct ocean interior acidification trends resolves—by definition—the perturbation of the marine CO_2_ system by the accumulation of anthropogenic carbon and assumes time-invariant ocean physics through its projection on a fixed density climatology. Hence, changes in the marine CO_2_ system that might locally be induced by changes in biogeochemical processes and their interplay with water mass transport are not resolved. In the “Caveats and limitations” section, we discuss the role of neglecting these processes.

For the interpretation and attribution of the OA trends, we report the sensitivity of the marine CO_2_ system parameters to a DIC perturbation of 1 μmol kg^−1^. We refer to these sensitivities as ΔΩ_arag_/ΔDIC and ΔH^+^/ΔDIC. In contrast to the corresponding sensitivities (βDIC and ωDIC) that were previously defined (*80*), we report sensitivities in absolute and not in relative terms.

### Definition of ocean regions, depth layers, and the seafloor

We aggregate our ocean interior acidification trends in the horizontal dimension based on Longhurst ocean provinces (*81*), which we combine into five large ocean regions (Fig. 2L and fig. S17) that match primary global patterns in our OA trends. We further average acidification trends over distinct depth layers with boundaries at 100-, 500-, 1500-, and 3000-m water depth. To constrain the acidification at the seafloor, we analyze the changes that occurred in the deepest layer of our OA reconstruction (determined by the underlying predictor fields) and above or equal to a depth limit of 500 m.

### Determination of uncertainty

We consider two main contributions to the uncertainty of our ocean interior acidification and sensitivity estimates: (i) the uncertainty associated with the observation-based reconstructions of the C_ant_ accumulation and (ii) the uncertainty of the modern-day climatologies of DIC, TA, salinity, and temperature.

To determine (i) the uncertainty contribution from the observation-based reconstructions of the C_ant_ accumulation, we adapt the same procedure developed for the C_ant_ reconstruction itself (*15*). This approach relies on an ensemble of 10 ΔC_ant_ reconstructions for 1994–2004 and 2004–2014 that were obtained on the basis of modified configurations of the eMLR(C*) method. The members of this ensemble capture the uncertainty of the ΔC_ant_ estimates related to data coverage and quality, the regional clustering of the observations, and other methodological aspects. For each ΔC_ant_ ensemble member, we computed the state of CO_2_ system individually for the reference years 1994, 2004, and 2014. Following the procedure developed for the C_ant_ reconstruction (*15*), we determine the C_ant_-induced uncertainty in the change of any CO_2_ system variable as the root of the sum of squares (RSS) of the differences between a standard case and the other nine ensemble members. For this purpose, we use the same standard case reconstruction of ΔC_ant_ and otherwise identical procedures as developed for the C_ant_ reconstruction (*15*), except for excluding one ensemble member that considers non-anthropogenic carbon concentration trends at the surface. Lacking a similar ensemble of reconstructions to derive a spatially resolved C_ant_-induced uncertainty for the period 1800–1994, we assume that these uncertainties are twice those of the 1994–2014 period, reflecting a factor of two between the global mean surface ΔC_ant_ of the two periods. For acidification estimates, the C_ant_ reconstruction uncertainties of each period are combined forward in time as the cumulative RSS, reflecting the increase of the uncertainty in the total change over time. Uncertainties for volumetric analysis are not accumulated over time. For sensitivity estimates, the C_ant_ reconstruction uncertainties of each period are combined forward in time as the cumulative RSS for reference years after 2002 (the reference year of the DIC/TA climatology) and backward in time for years prior 2002.

To determine (ii) the uncertainty contribution from modern-day climatologies of DIC and TA, we computed the CO_2_ system from modified fields of these climatologies. Specifically, we increased/decreased the values of DIC and TA by their corresponding mapping errors (up to ~50 μmol kg^−1^) provided together with the climatologies (*50*). In addition, we computed the CO_2_ with alternative climatologies for salinity and temperature from GLODAP. We determined the combined uncertainty contribution from the climatologies as the mean absolute offset between these five alternative reconstructions and those obtained from our default choice of unperturbed climatologies. It should be noted that the mapping uncertainties in the carbonate climatologies tend to be high in regions of low sampling density [see figures 9 and 10 in (*50*)], for example, the Southern Ocean. This mapping uncertainty is propagated forward to our acidification uncertainties. The uncertainty components (i) and (ii) were combined as the RSS of the two individual contributions and reported along with the trends in the CO_2_ system variables obtained from the standard case reconstruction of ΔC_ant_ and the original modern-day DIC/TA climatologies.

In addition to the formal uncertainty quantification described above, we also present acidification estimates that consider ocean interior temperature changes from 1994 to 2004 and 2004 to 2014. For this purpose, we computed the changes in temperature for both decades from version 3 of the observation-based ocean interior temperature reconstruction from Institute of Atmospheric Physics (IAP) (*75*) and added these decadal trends to the temperature climatologies before computing the state of the marine CO_2_ system. The decadal acidification trends that are obtained differ marginally from those without the temperature trends and are displayed in fig. S2 as global and regional mean profiles.
