# Supplementary material for: Progression of ocean interior acidification over the industrial era
Source: Sci Adv. 2024 Nov 27;10(48):eado3103. doi: 10.1126/sciadv.ado3103 (PMC11601202; doi:10.1126/sciadv.ado3103)
Supplement: Supplementary file 1 — Figs. S1 to S17 [file sciadv.ado3103_sm.pdf]

Science Advances
AAAS

Supplementary Materials for

Progression of ocean interior acidification over the industrial era

Jens D. Müller and Nicolas Gruber

Corresponding author: Jens D. Müller, jensdaniel.mueller@usys.ethz.ch

Sci. Adv. **10**, eado3103 (2024)
DOI: 10.1126/sciadv.ado3103

This PDF file includes:

Figs. S1 to S17

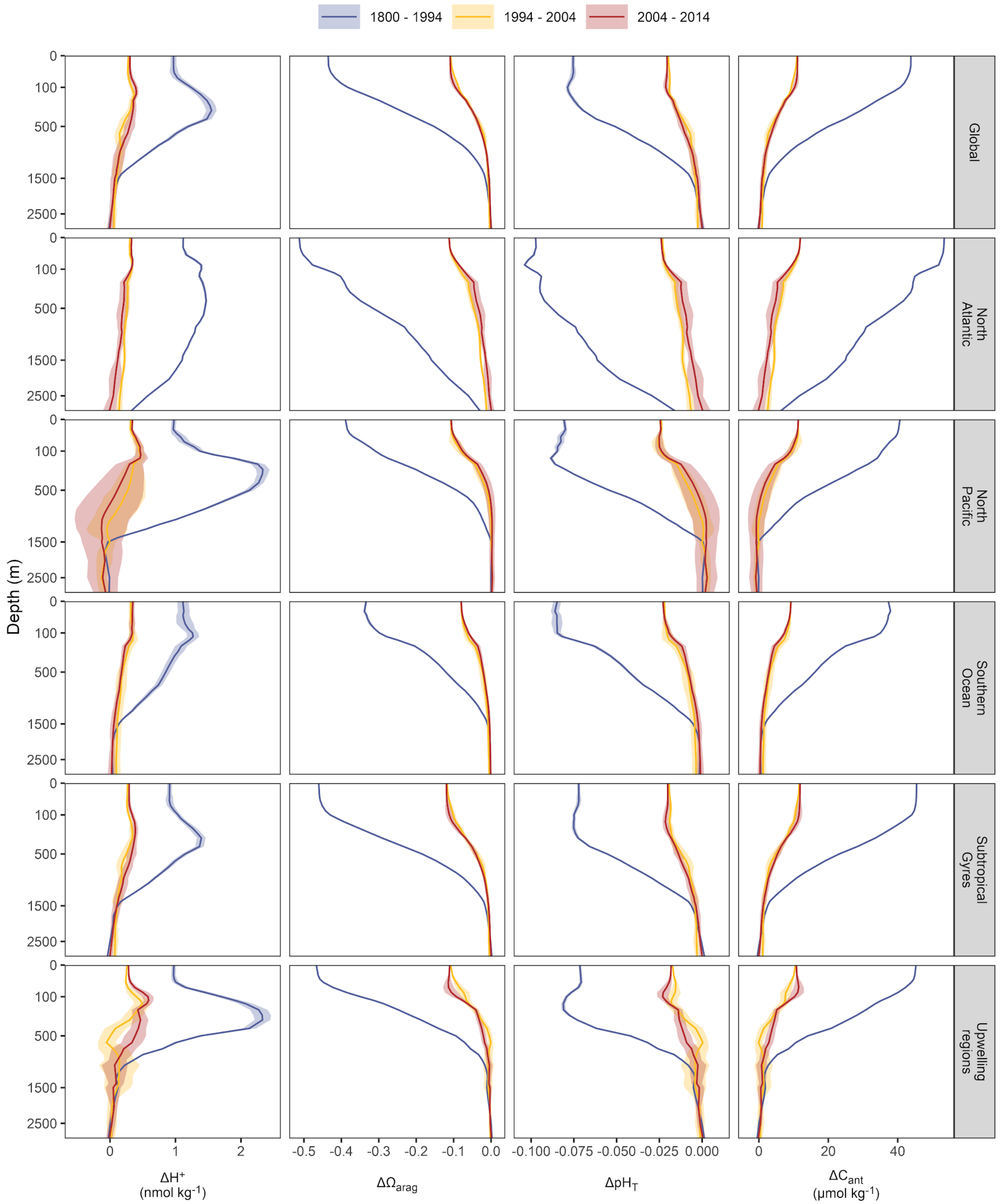

Fig. S1: Same as Fig. 1, but showing incremental changes between reference years instead of total changes since 1800. CO_2 system sensitivities are not displayed.

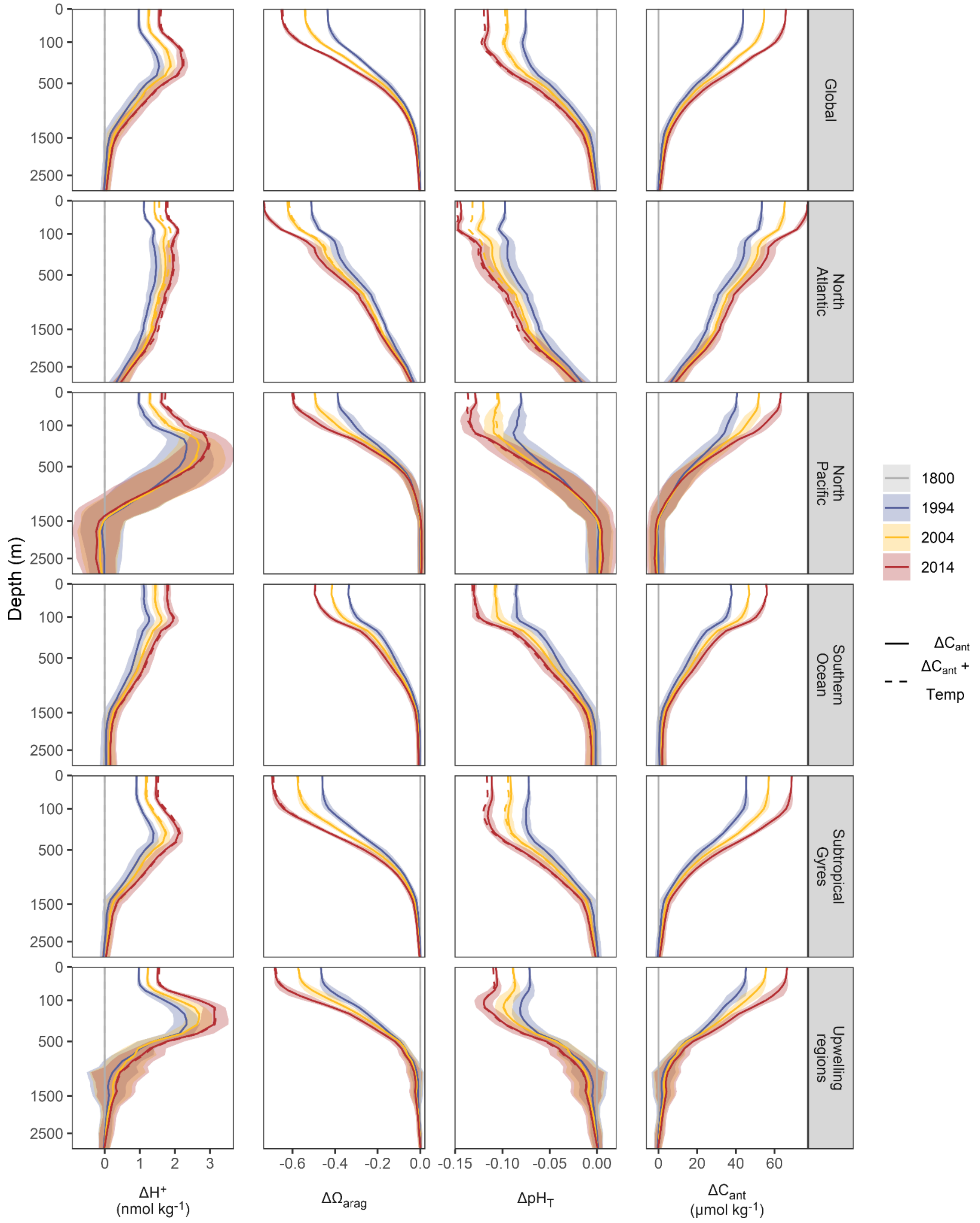

Fig. S2: Same as Fig. 1, but averaged across individual ocean biomes according to Fig. S17. In addition to the acidification trends driven solely by the accumulation of C_{ant} (solid lines), we consider here also the impact of observation-based temperature changes for the past two decades (dashed lines). Note that temperature-induced changes in natural carbon (e.g. DIC loss due to warming) are not considered.

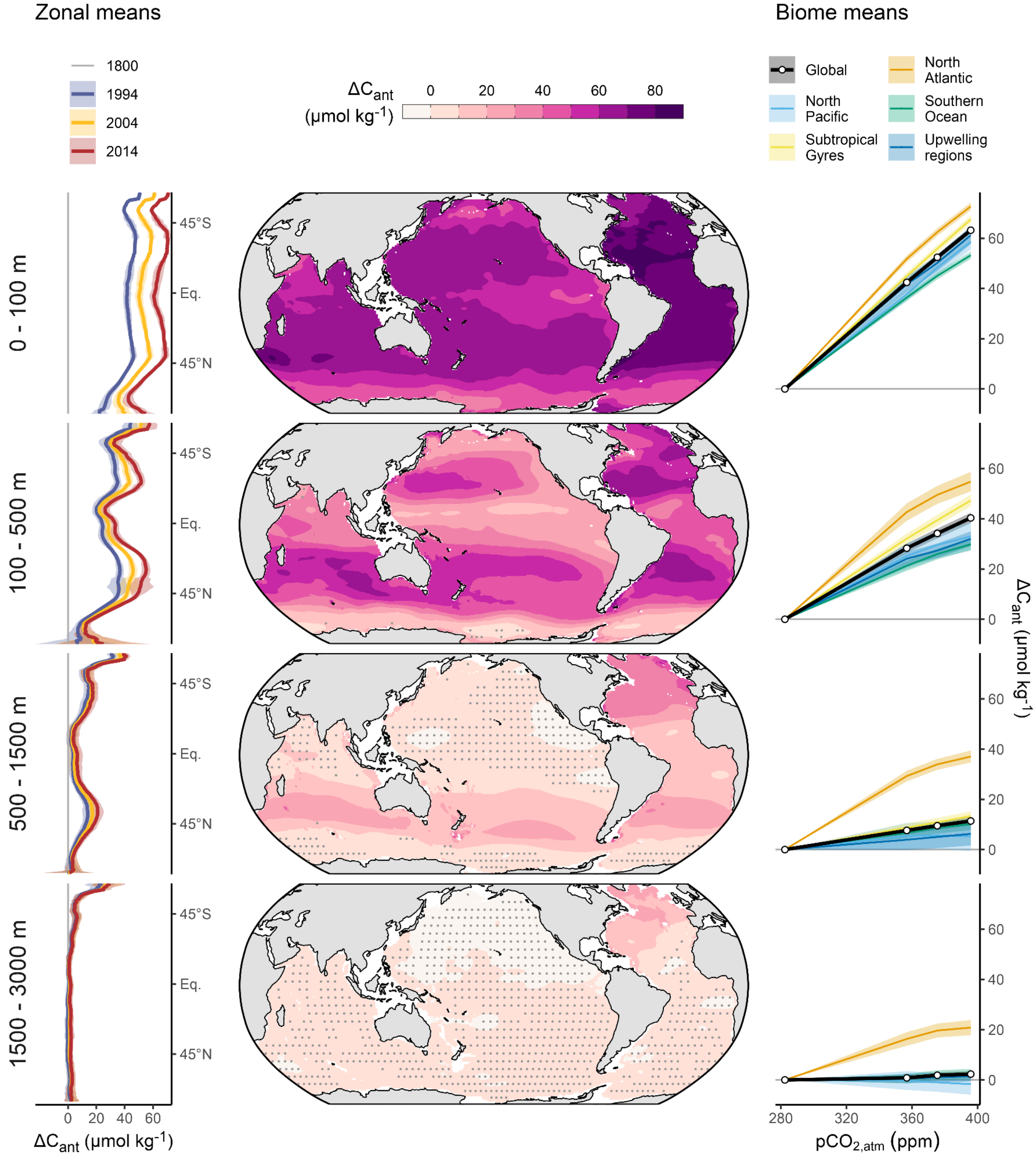

Fig. S3: Same as Fig. 2, but for changes in the anthropogenic carbon content (ΔC_{ant}).

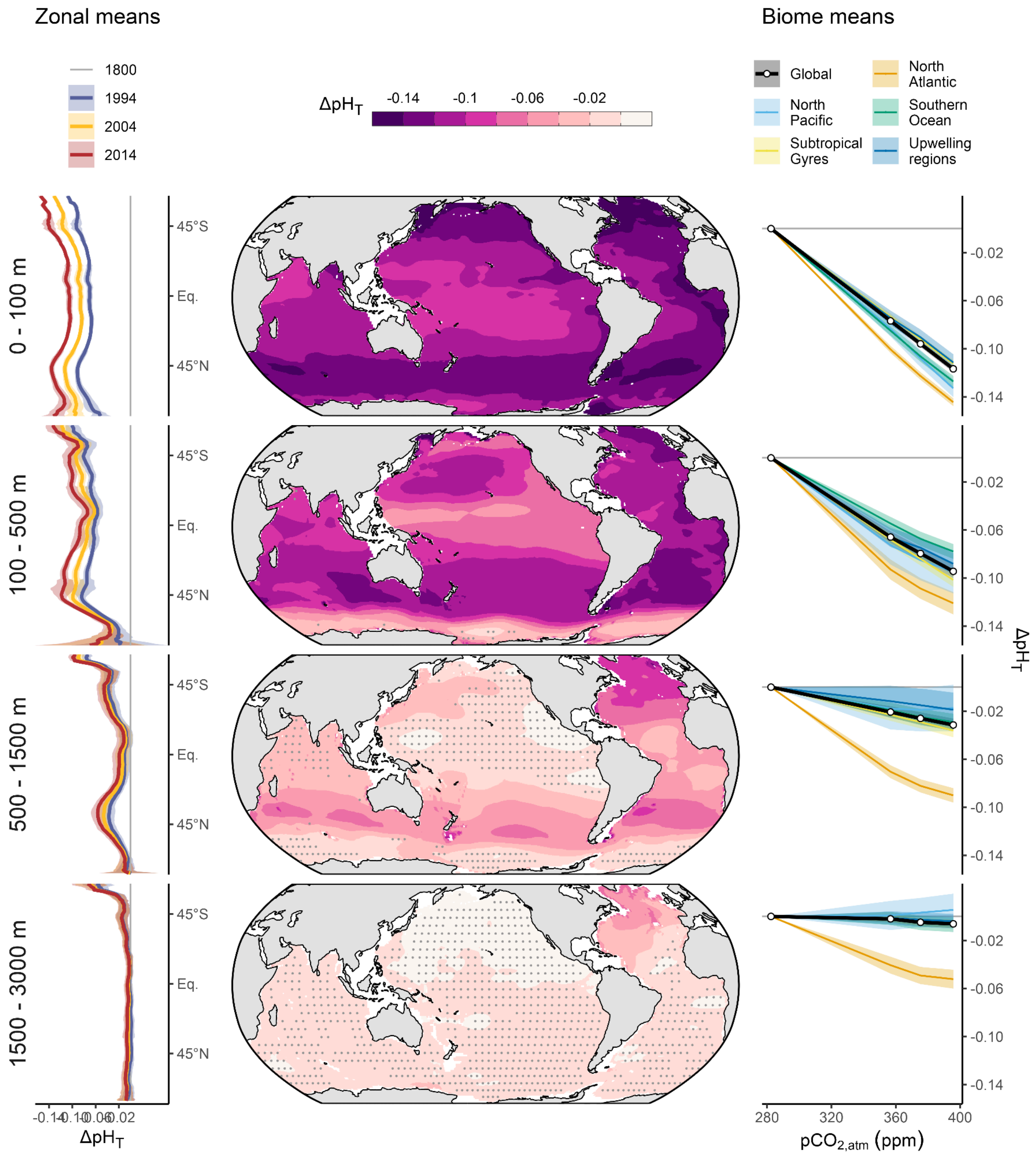

Fig. S4: Same as Fig. 2, but for changes in pH on the total scale (ΔpH_T).

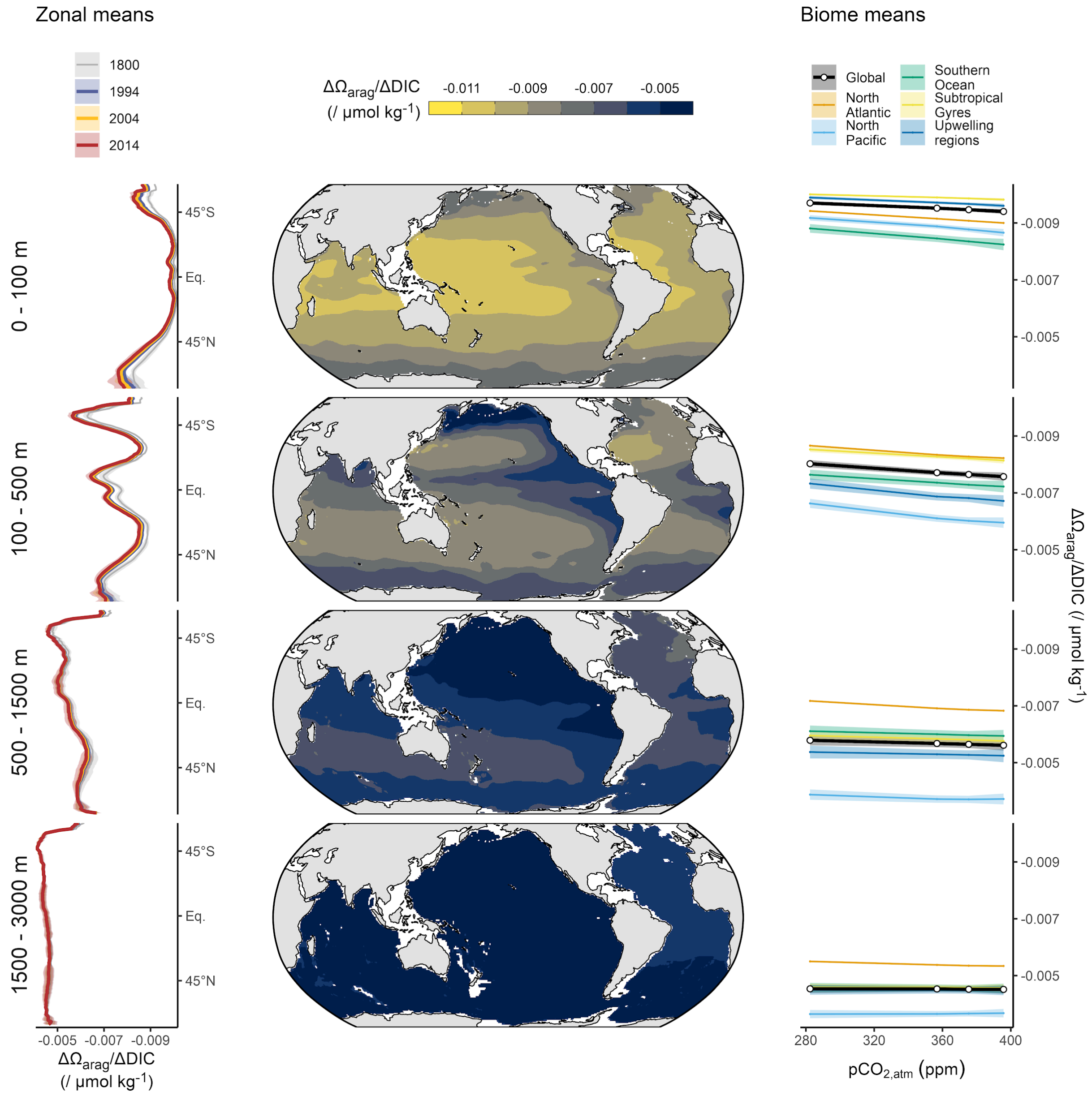

Fig. S5: Sensitivity of the saturation state of aragonite to a change in the dissolved inorganic carbon concentration ($\Delta\Omega_{arag}/\Delta DIC$) averaged over four depth layers (rows). Central panels show maps of $\Delta\Omega_{arag}/\Delta DIC$ in 2014. Left panels show zonal mean $\Delta\Omega_{arag}/\Delta DIC$ for the reference years 1800, 1994, 2004 and 2014. Right panels show the global and regional mean $\Delta\Omega_{arag}/\Delta DIC$ as a function of the increase in atmospheric CO_2.

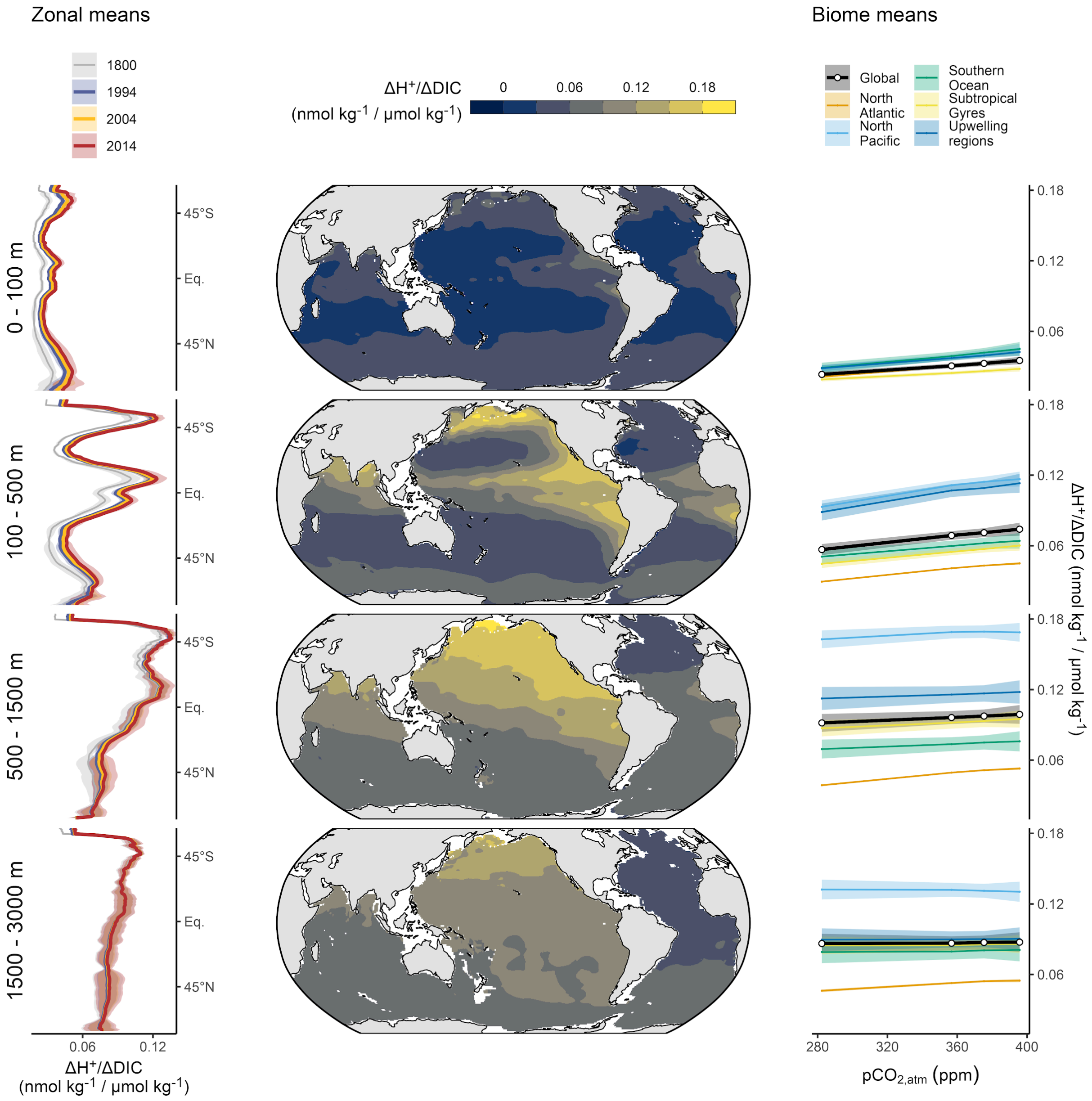

Fig. S6: Same as Fig. S5, but for the sensitivity of the free proton concentration to a change in the dissolved inorganic carbon concentration ($\Delta H^+/\Delta DIC$).

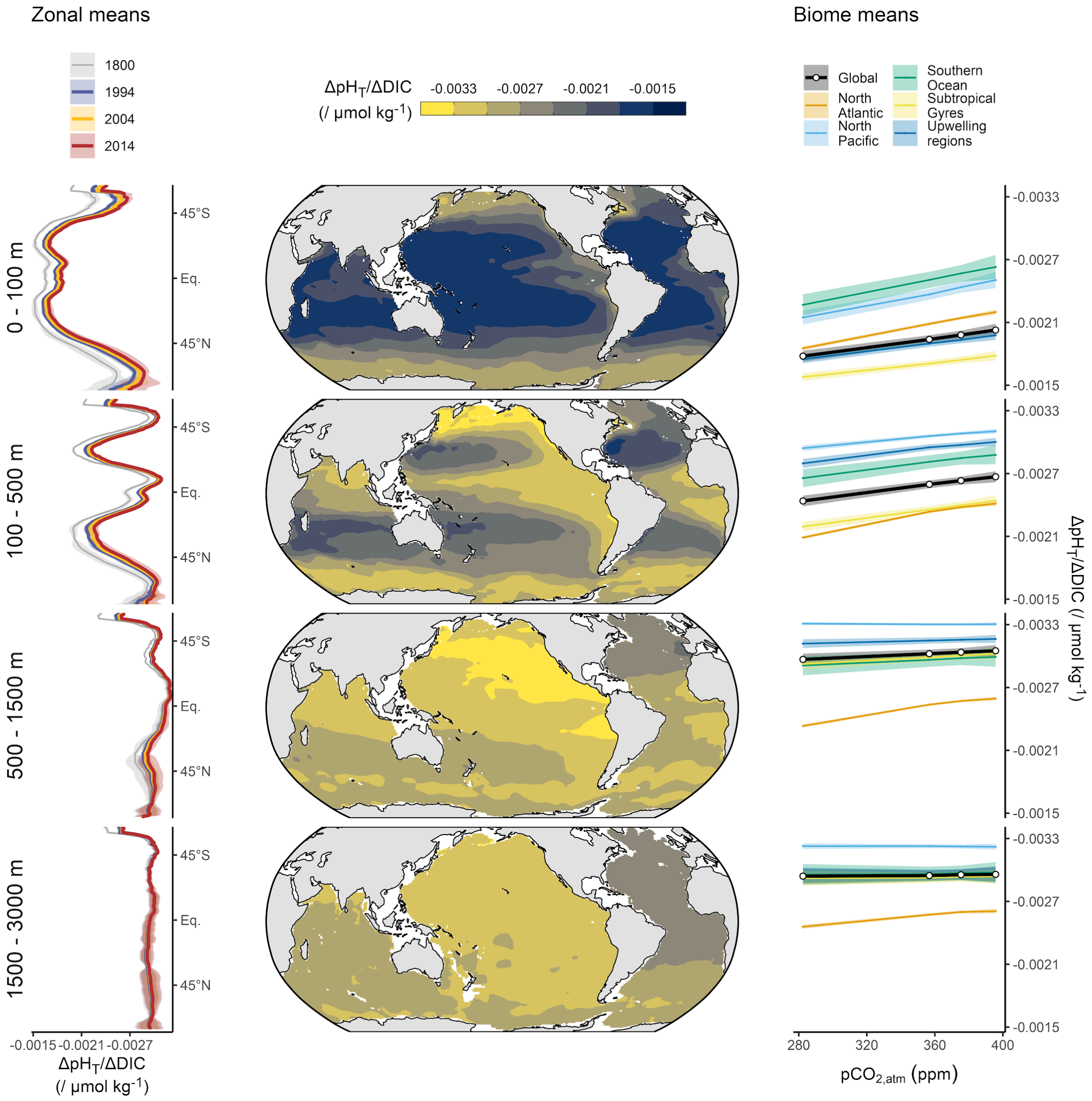

Fig. S7: Same as Fig. S5, but for the sensitivity of pH on the total scale to a change in the dissolved inorganic carbon concentration ($\Delta pH_T/\Delta DIC$).

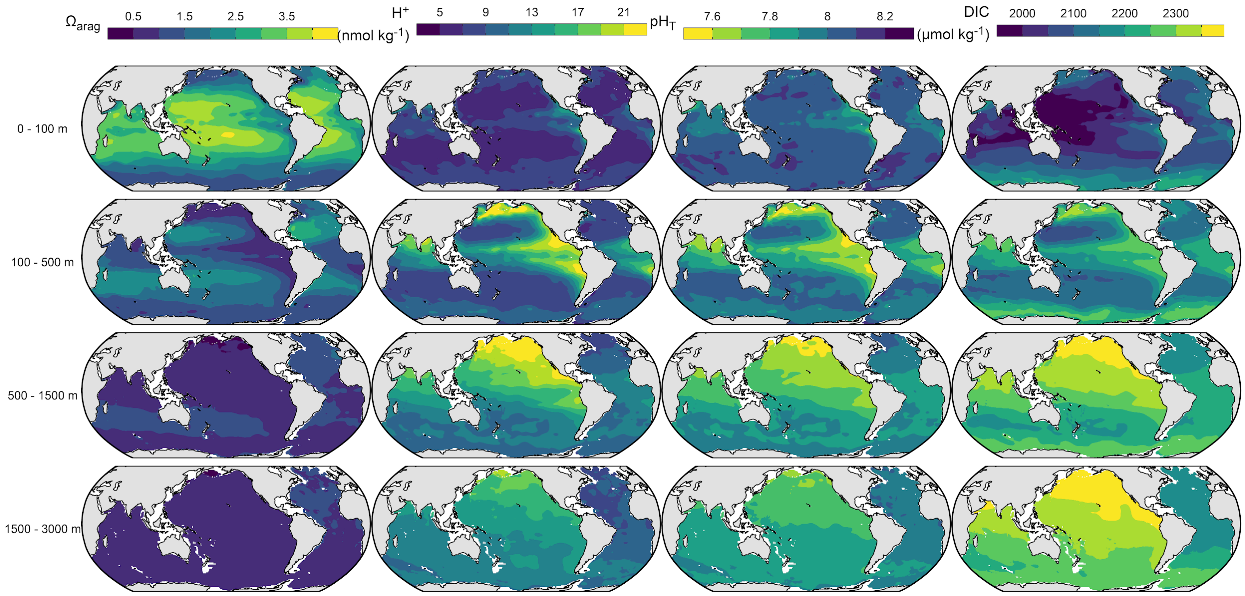

Fig. S8: Absolute levels of the saturation state of aragonite (Ω_{arag}), the free proton concentration (H^+), pH on the total scale (pH_T) and dissolved inorganic carbon concentration (DIC) in 2014 averaged over four depth layers (rows).

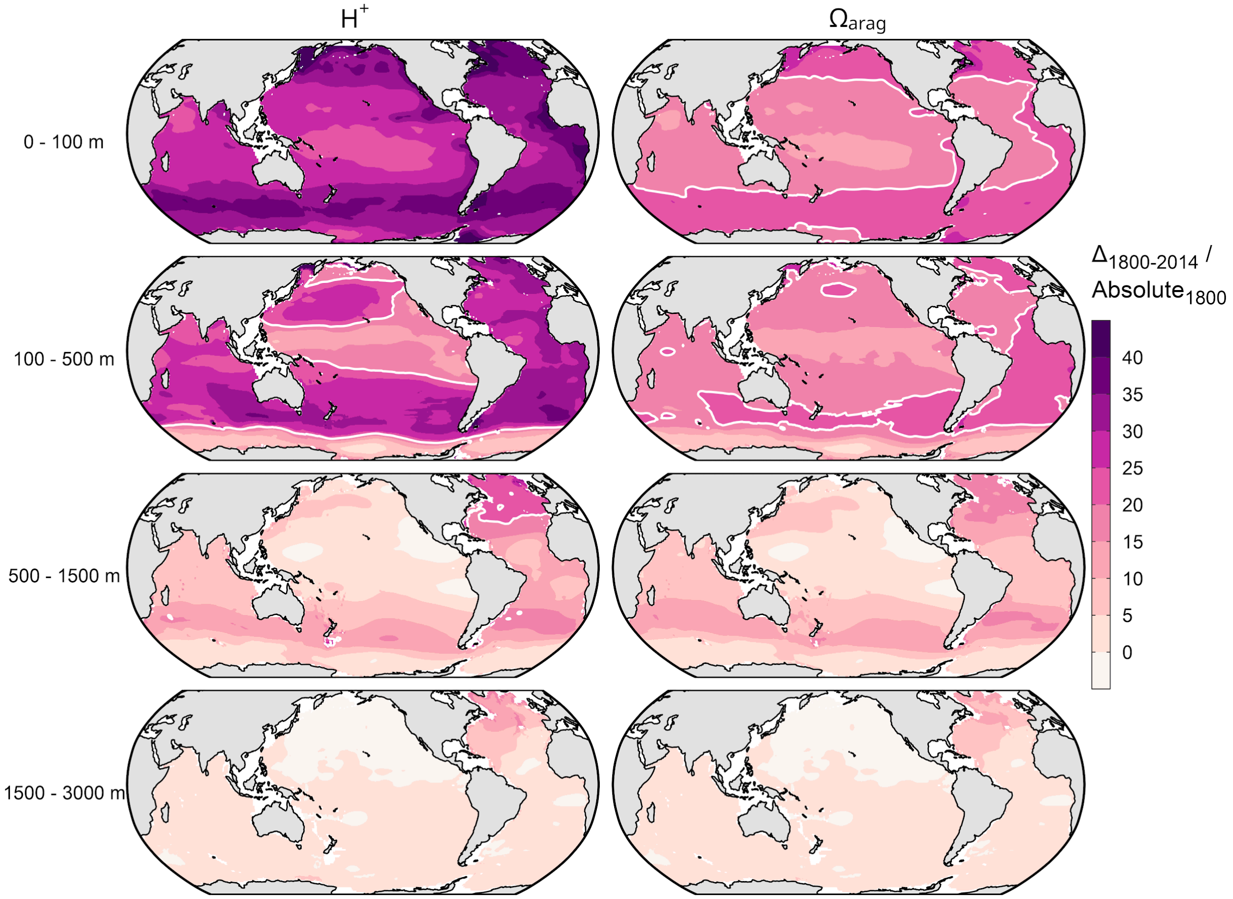

Fig. S9: Acidification trends over the industrial era (1800-2014) relative to the preindustrial state of the free proton content (ΔH^+, left panels) and the saturation state of aragonite ($\Delta\Omega_{arag}$, right panels) averaged over four depth layers (rows). White contour lines highlight relative changes of 30%.

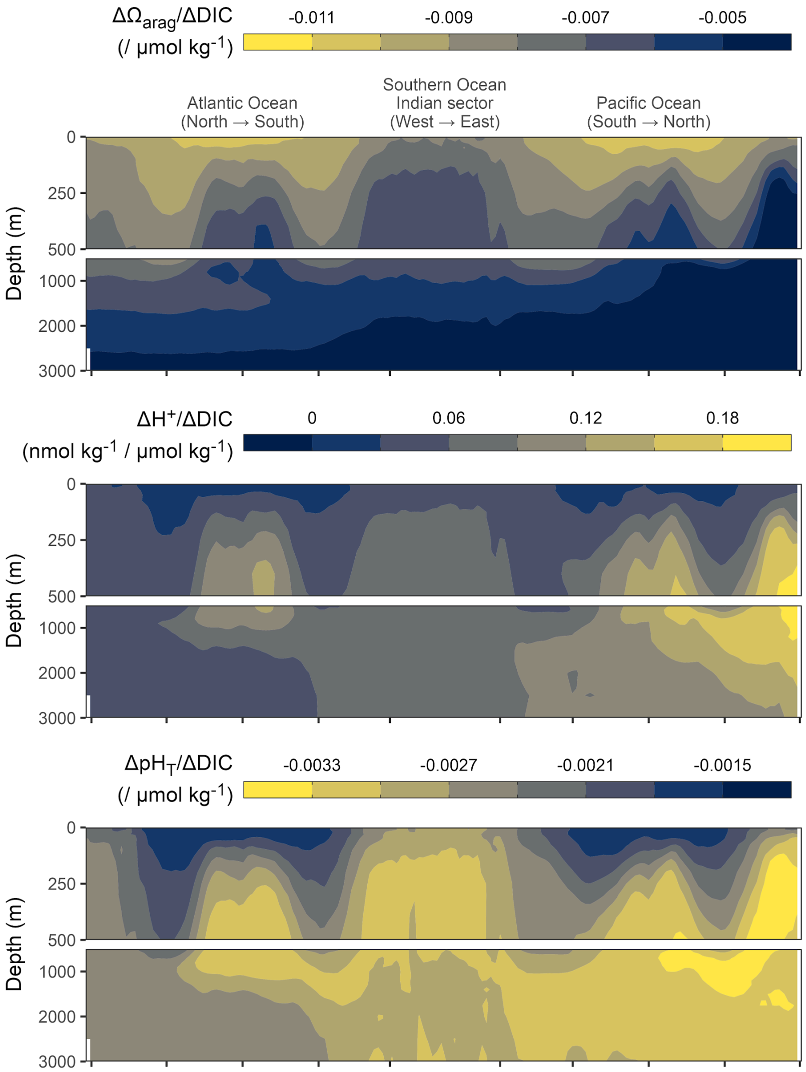

Fig. S10: Global mean sections of the sensitivity of the saturation state of aragonite ($\Delta\Omega_{arag}$), the free proton content (ΔH^{+}), and pH on the total scale (pH_T) to a change in the dissolved inorganic carbon content (ΔDIC).

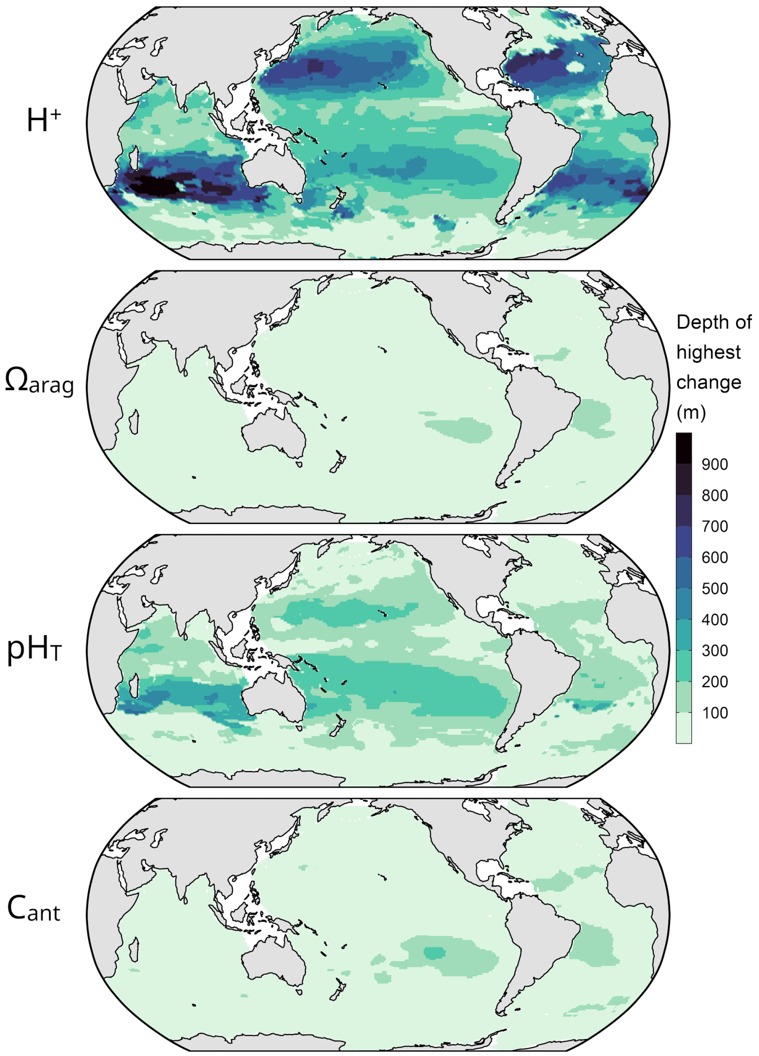

Fig. S11: Depth of the highest absolute changes in the free proton concentration (H^+), the saturation state of aragonite (Ω_{arag}), pH on the total scale (pH_T) and anthropogenic carbon concentration (C_{ant}) from 1800 through 2014.

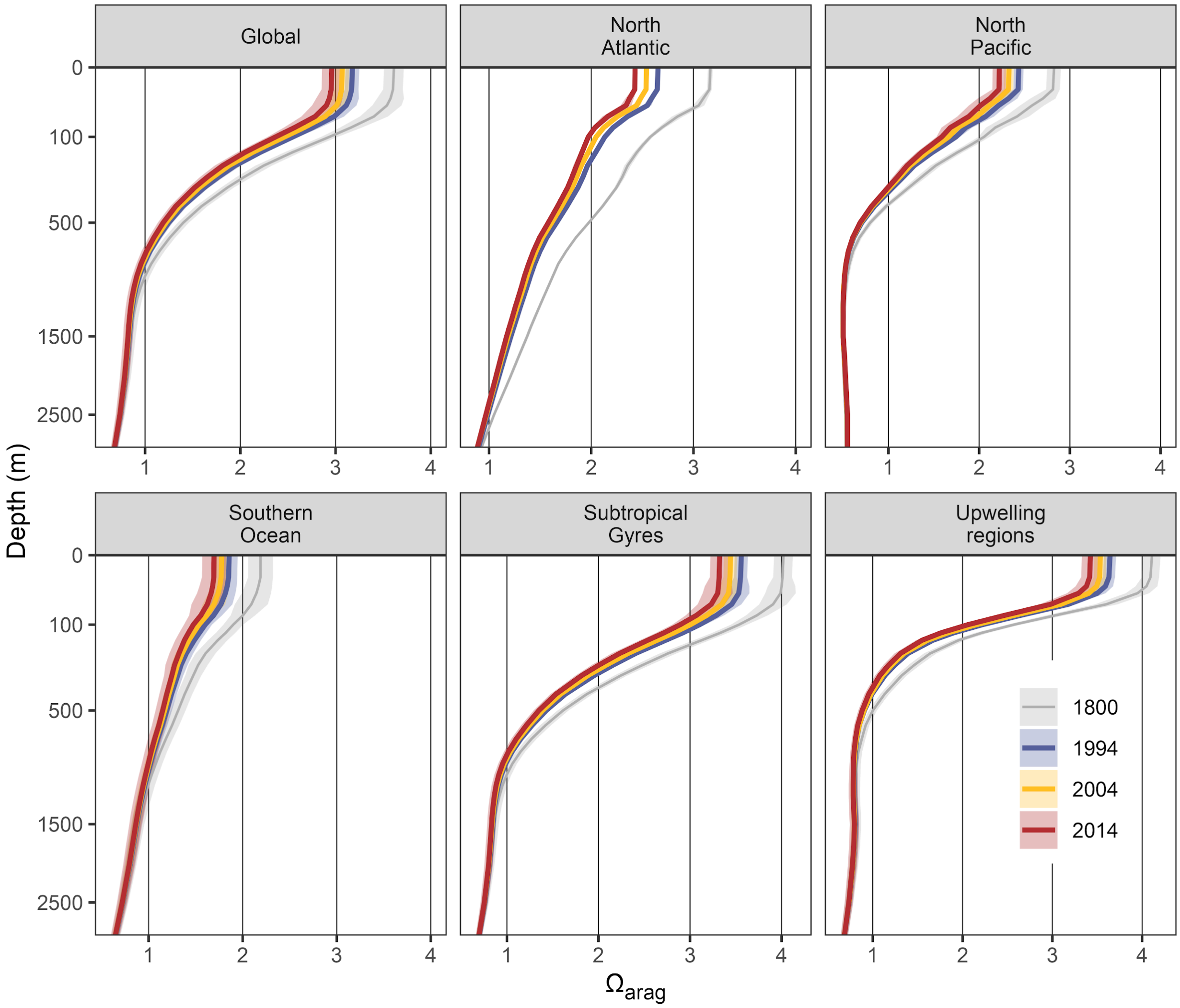

Fig. S12: Mean vertical profiles of the saturation state of aragonite (Ω_{arag}) averaged over five ocean regions (Fig. S17) and globally, showing absolute values for four reference years between 1800 and 2014.

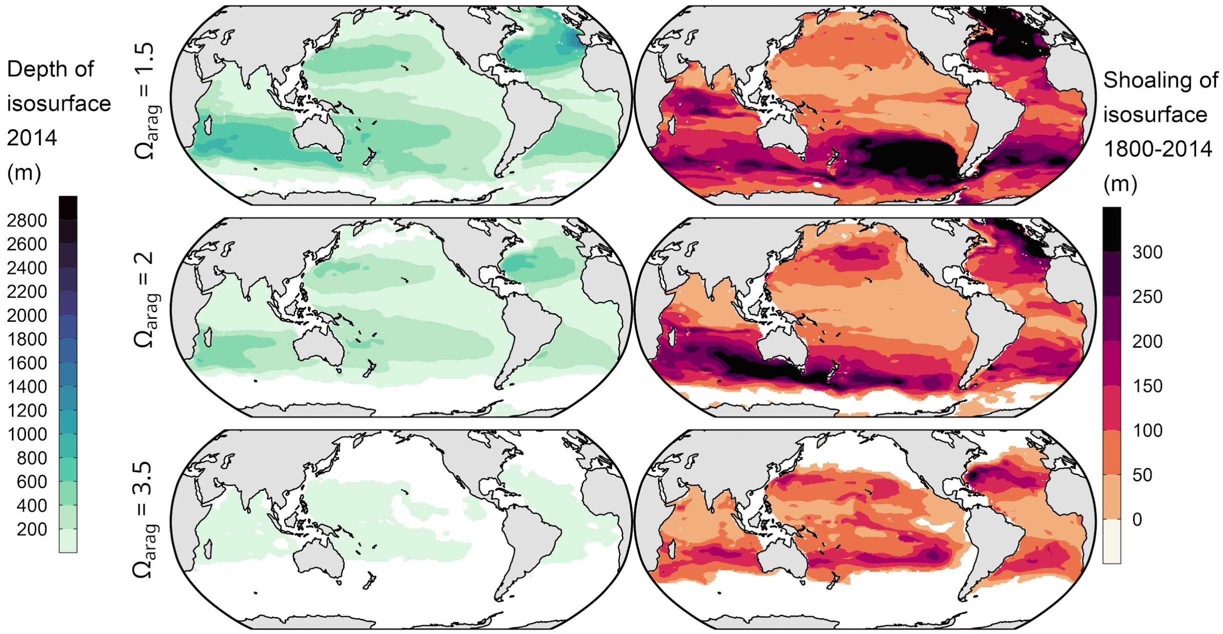

Fig. S13: Same as Fig.6, but for the saturation thresholds 1.5, 2, and 3.5.

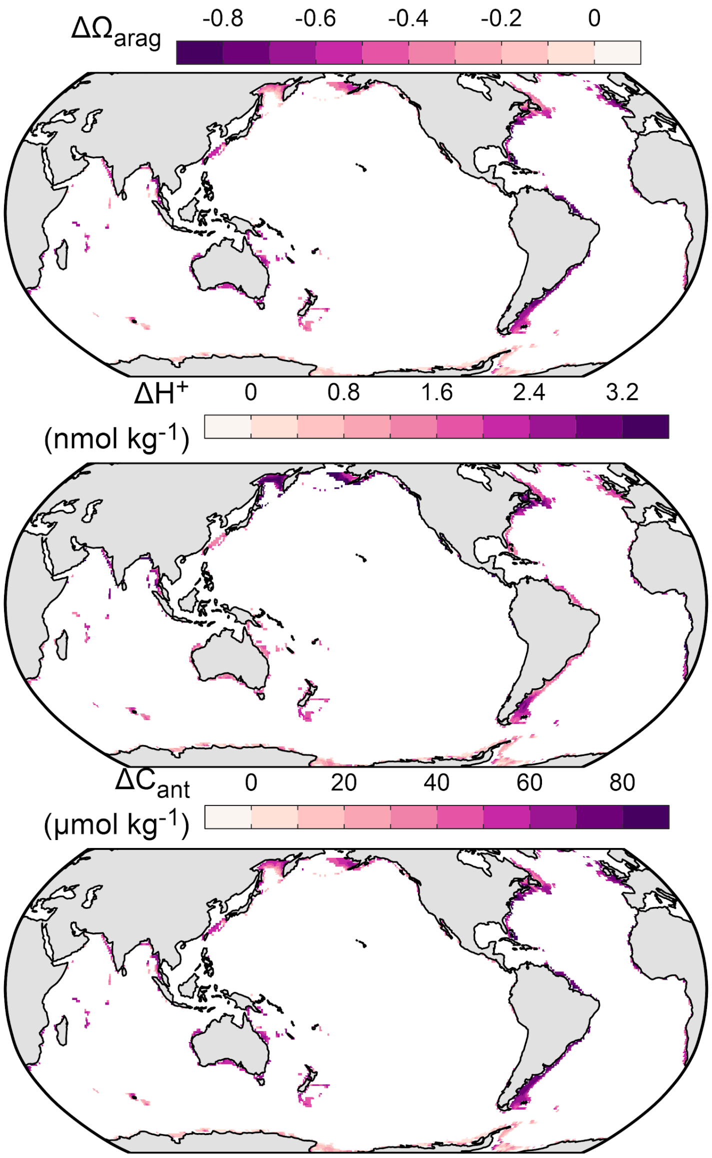

Fig. S14: Maps of acidification at the seafloor located within the top 500 m of the ocean. The areas shown here underlie also the results shown in Fig. 5. Changes are displayed for the saturation state of aragonite ($\Delta\Omega_{arag}$), free proton concentration (ΔH^{+}), and anthropogenic carbon concentration (ΔC_{ant}).

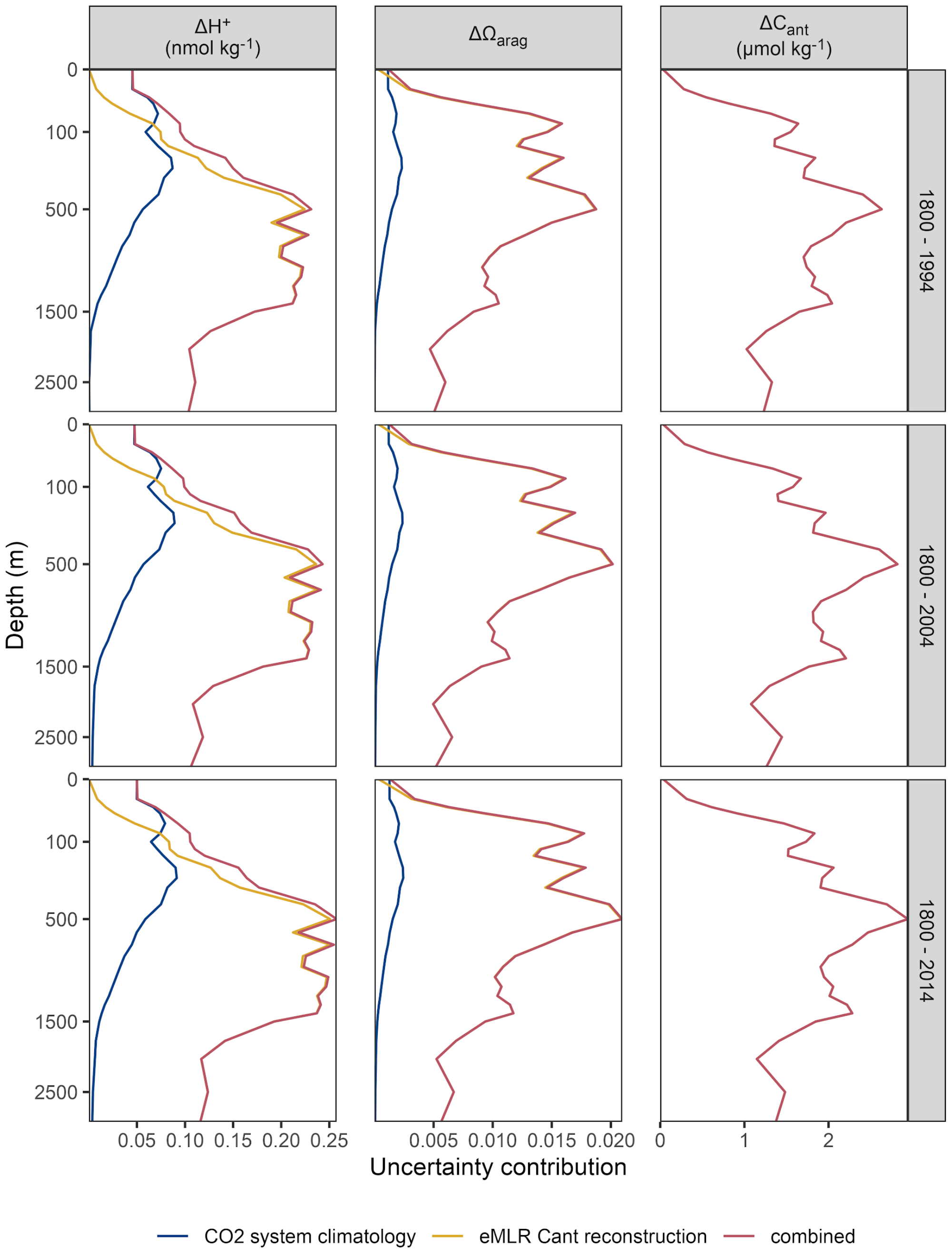

Fig. S15: Global mean vertical profiles of the contributions to the uncertainty in changes in the saturation state of aragonite ($\Delta\Omega_{arag}$), free proton concentrations (ΔH^+), and anthropogenic carbon concentration (ΔC_{ant}). Panel columns distinguish changes since 1800 for the reference years 1994, 2004, and 2014.

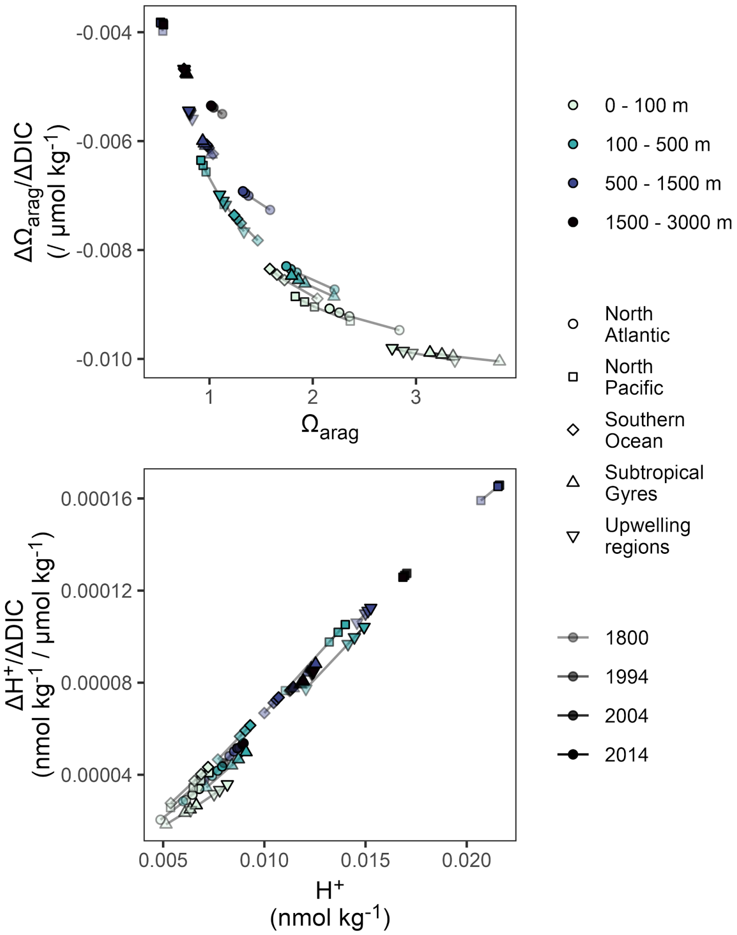

Fig. S16: Sensitivity of the free proton concentration (H^+) and the saturation state of aragonite (Ω_{arag}) to a change in DIC as a function of their absolute value. The relationship is displayed for the mean state of the CO_2 system across the 5 regions (symbol shape), four depth layers (color) and four reference years (transparency) analyzed in this study. Estimates of the same region and depth layer are connected by a line to emphasize the temporal evolution.

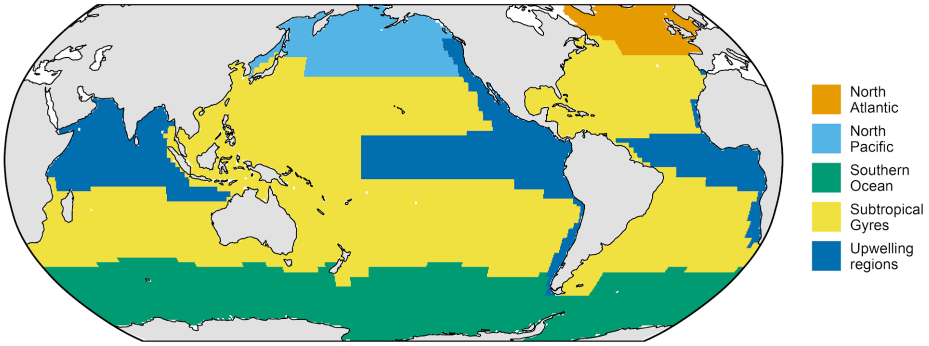

Fig. S17: Ocean regions used to average ocean acidification trends in the horizontal dimension, based on aggregated provinces from Longhurst (2007).
